# Intelligent surgical drainage - digitizing the analysis of drainage fluid in patients with surgical drains

**DOI:** 10.1371/journal.pone.0325072

**Published:** 2025-07-28

**Authors:** Anastasia Meckler, Sebastian Künert, Leonardo Poggi, Julia Jeske, Lukas Schipper, Thanusiah Selvamoorthy, Felix Nensa, Bernadette Hosters, Michael Fabian Berger, Ramsi Siaj, Mario Vincent Roser

**Affiliations:** 1 Department of Pediatric Surgery, University Hospital Essen, Germany; 2 Institute of Diagnostic and Interventional Radiology and Neuroradiology, University Hospital Essen, Germany; 3 Institute for Artificial Intelligence in Medicine (IKIM), University Hospital Essen, Germany; 4 Institute for Medical Informatics, Biometry and Epidemiology, University of Duisburg-Essen, Germany; 5 Department of Nursing Development and Nursing Research, University Hospital Essen, Germany; 6 Elixion Medical GmbH, Düsseldorf, Germany; University of Health and Allied Sciences, GHANA

## Abstract

Surgical drains are essential in post-operative care, where timely removal is critical to prevent complications. Early removal can result in seromas and hematomas, while delayed removal may lead to infections. Traditional manual analysis of drain output is time-consuming and often unreliable, necessitating a shift towards digital methods. This study used a compact mini-spectrometer to analyze surgical drain output quickly and non-invasively. The spectrometer operates in the 340–850 nm range with 288 discrete detection channels. A total of 528 samples were collected from 181 patients aged 0–85 years. Fourteen laboratory parameters, including albumin, amylase, bilirubin, total protein, LDH, lipase, erythrocytes, hemoglobin, and triglycerides were analyzed. Notable correlations were observed for several parameters. This study employed correlation, regression and classification analyses to investigate the relationships between various biochemical laboratory parameters in drain output and their absorption peaks at specific wavelengths. The data obtained from standard procedures in a certified central laboratory were compared with data collected using the mini-spectrometer. Significant correlations were found, particularly for hemoglobin and erythrocytes at 586 nm (r = −0.67 and r = −0.46, respectively). Hemoglobin also correlated with wavelengths at 514 nm (r = −0.62) and 557 nm (r = −0.45). Bilirubin showed peaks at 582 nm (r = 0.56) and 496 nm (r = −0.49). Regression and classification models, incorporating random effects, provided enhanced performance. The classification models effectively differentiated between pathological and non-pathological values, with hemoglobin showing an area under the curve (AUC) of 0.947 and a Balanced Accuracy (BAC) of 0.853. Triglycerides had an AUC of 0.941 and a BAC of 0.789. Models for LDH, bilirubin, and erythrocytes also achieved AUC values over 0.9, with BAC values exceeding 0.79. This study demonstrates the potential of mini-spectrometers integrated into surgical drains to improve post-operative drainage management, potentially offering faster, more reliable analyses compared to traditional methods.

## Introduction

The benefits of using surgical drains in the post-operative management of patients highly depend on the right retention time before the drain is removed [1,2]. Removing a drain too early can lead to seroma and hematoma formation [3–6], while a delayed removal poses the risk of ascending infections [7–9]. Commonly used criteria for determining the timing of drain removal include the volume and quality of the drainage output [10]. Traditionally, analysing drainage fluid is a time-consuming process that often yields inconsistent results. It involves manually measuring the volume, visually assessing the fluid quality, or sending samples for laboratory analysis [11,12]. This approach can result in delays in identifying potential complications such as infections or internal bleeding, underscoring the need for more efficient and accurate monitoring methods [13].

We recently introduced a novel medical device based on a compact mini-spectrometer which can analyze surgical drainage fluid both quantitatively and qualitatively [14]. Our prototype is an innovative system which combines intelligent algorithms and digitalization to enhance the monitoring and management of postoperative drainage. In this study here, as a proof-of-concept, we analyzed surgical fluid drainage sampled after abdominal surgery in an experimental setting.

The goal of our study was to train our prototype and build a robust statistical model capable of predicting laboratory parameters using the measured spectra to detect changes in a large population of fluid samples.

## Materials and methods

### Prototype development

In this study we aimed at characterizing surgical drainage fluid with a sophisticated yet affordable and compact mini-spectrometer. The spectrometer used in our prototype offers fast and non-invasive measurement in the detection range between 340 nm until 850 nm with light intensity values ranging from a minimum of 0 until a maximum of 65000 (unitless) being captured by 288 discrete detection channels. It thus exceeds the perception of the human observer at both ends of the visible spectrum (roughly 380 nm until 700 nm) [15].

**Fig 1:**
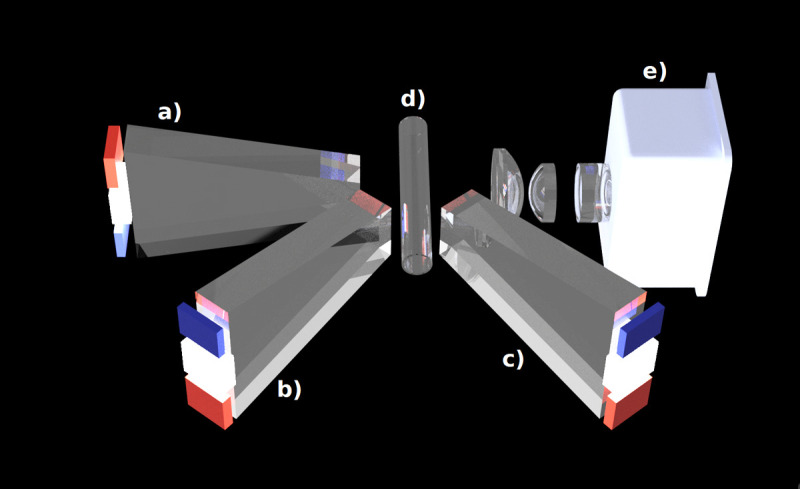
The main components of the mini spectrometer. The spectrometer head **(e)** directs light through a lens array towards the sample held in a tubing chamber **(d)**. The interaction between the sample and the incoming light is captured from three different angles: direct transmission **(a)**, angular transmission **(b)**, and angular reflection **(c)**.

The mini-spectrometer was integrated with a custom lens system and a controlled light source to ensure broad-spectrum illumination (see Fig 1). Each sample was illuminated from three different angles: DT (direct transmission), AT (angular transmission), and AR (angular reflection). Initially, two exposure times were tested for each angle. However, some exposure settings led to overexposure of the samples. To ensure clear and accurate data, only one optimal exposure time was selected for each illumination setting (see Table 1). In total this amounts to a cuboidal data structure of 288 x 3 = 864 light intensity values influenced by the absorption of the biochemical properties of the fluid sample.

**Table 1:** Light pathways with exposure times.

	Direct transmission (DT)	Angular transmission (AT)	Angular reflection (AR)
**Exposure time**	20 μs	200 μs	320 μs

A first evaluation platform was built using FDM 3D-printing and PLA as printing material. This resulted in unreliable positioning of the optical lens array as well as the illumination source due to the flexible nature of PLA and relatively low resolution of FDM 3D-printing. An improved evaluation platform was built which included numerous improvements such as mechanical isolation of critical parts as well as using a much more rigid resin 3D-printing material and high-resolution SLA 3D-print for all optical components.

### Study population

**Fig 2:**
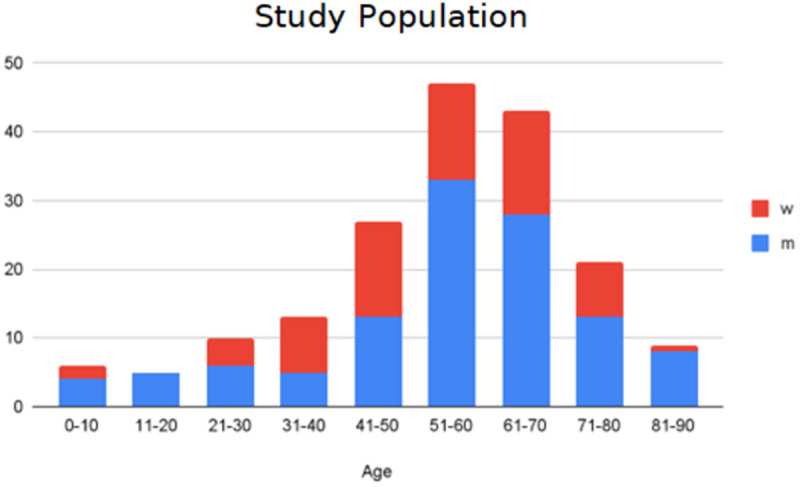
The overview of age and gender distribution of Study Population. The chart shows the age distribution of the study population across different age groups from 0 to 90 years, broken down by gender (blue: m = male, red: w = female). The X-axis displays the age groups in 10-year intervals from 0–10 to 81–90. The Y-axis represents the number of individuals.

In this study, patients ranging in age from 0 years to 85 years participated from the patient population of the Department of Pediatric Surgery as well as the Department of General, Visceral, and Transplant Surgery at the University Hospital Essen. We collected 528 samples from 181 patients. The overview of age and gender distribution can be seen in Fig 2. To participate in the study, written consent from adult patients is required. For minor patients, written consent from their legal guardians as well as assent from the minors, when possible, themselves were obtained. The study received ethical approval from the Ethics Committee of the Medical Faculty at the University of Duisburg-Essen under reference number 21–10407-BO.

### Sample collection and measurements

Fourteen different laboratory parameters were chosen for examination in drainage samples. These parameters included albumin, amylase (pancreatic), bilirubin, total protein, glucose, uric acid, LDH, lipase, erythrocyte, hemoglobin, leukocytes, mononuclear cells, polymorphonuclear cells and triglycerides.

The samples were collected from the patient’s surgical drain at the bedside between 11.04.2022 and 01.02.2023. Typical drain types were Robinson drains, Redon drains, and Easy-Flow drains. Each sample was divided into two aliquots: Aliquot 1 for measurement in the central laboratory (one reaction tube each with and without EDTA), Aliquot 2 for measurement with the mini-spectrometer (15 ml reaction tube). All samples were immediately frozen and stored at −80°C until further processing. Aliquot 1 (for the central laboratory at the University Hospital Essen) was transported on dry ice. After thawing, the concentration of each parameter was measured using standard central laboratory methods. Aliquots 2 (for spectrometric measurement) were thawed immediately before use and measured by the prototype. After each measurement, the drainage sample was discarded, and the tubes and measurement chamber of the prototype were thoroughly flushed with sterile water to prevent cross-contamination between samples. After completing the measurements on each respective day, the prototype was flushed with isopropyl alcohol to ensure the prevention of sample cross-contamination and bacterial growth.

Some of the samples were used for preliminary analysis to select the optimal wavelengths. Additionally, in some cases, there was insufficient material available, or the sample was otherwise unsuitable for laboratory measurement. Hence, the number of samples that underwent the entire analysis process varies between 420 and 454, depending on the parameters investigated (see Table 2). Laboratory values and spectral data are presented in S3 Table.

**Table 2:** Overview of drainage parameters, cut-off values, and sample counts per parameter.

Drain Marker	Cut-off value	Nr. samples	Pathological samples in %	Healthy samples in %
Triglycerides	200 mg/dl	453	3,8	96,2
Uric acid	7.2 mg/dl	453	8,3	91,7
Albumin	2.5 mg/dl	449	16,0	84,0
Amylase	53 U/l	447	19,4	80,6
Bilirubin	1.2 mg/dl	453	19,4	80,6
Lipase	53 U/l	454	28,6	71,4
Glucose	50 mg/dl	453	41,9	58,1
Total protein	2.5 g/dl	454	50,3	49,7
LDH	247 U/I	449	64,9	35,1
Hemoglobin	0 mg/dl	425	66,7	33,3
Erythrocytes count	0	427	86,2	13,8
Mononuclear cells	0	420	91,7	8,3
Polymorphonuclear cells	0	420	98,0	2,0
Leucocytes	0/nl	419	99,0	1,0

In Table 2, an overview of all drainage parameters is provided. For the classification analysis, the values had to be divided into pathological and non-pathological values. Since there are no predefined boundaries for drainage parameters, cut-off ranges were adopted from the performance directory of the central laboratory of the University Hospital Essen (Version 1.4), which apply to blood values. However, for the markers hemoglobin and erythrocytes, the threshold for pathology was set to 0 mg/dl, as the presence of these markers in surgical drain fluids could indicate a source of bleeding. Additionally, albumin and total protein were set at the cut-off value of 2,5 mg/dl, which would be important for the diagnosis of ascitic punctures [16].

A sample is categorized as pathological if its value is less than or equal to the corresponding cut-off value; otherwise, it is classified as healthy. Additionally, the percentage of pathological and healthy samples was calculated.

### Statistical analysis

As part of the present work we build a solid statistical model able to determine the laboratory value of the considered parameters on the base of the measured spectra.

#### Spectra normalization.

The correct calibration of the spectrometer is crucial for the acquisition of clean data. Nevertheless, artifacts caused by external events can greatly affect the measured light intensities [17]. When comparing different spectra, pronounced scattering in the intensity values becomes evident. This creates a bias in the input data that can negatively impact the outcome of statistical analysis methods [17]. In the present work, we correct for such phenomena by scaling the spectra using the Standard Normal Variate (SNV) method. The SNV method transforms a spectrum $\vec{s}$ into a new spectrum $\vec{\tilde{s}}$ by mapping each datapoint $s_{i}\in \vec{s}\;$ to a new datapoint $\tilde{s_{i}}\in \vec{\tilde{s}}\;$ using the following equation: $\tilde{s_{i}}=\frac{s_{i}-\mu (\;\vec{s})}{\sigma (\;\vec{s})}$, where $\mu (\;\vec{s})$ and $\sigma (\;\vec{s})$ are, respectively, mean and standard deviation of the original spectrum $\vec{s}$. As a consequence, the transformed spectrum $\vec{\tilde{s}}$ has zero mean and unit variance.

**Fig 3:**
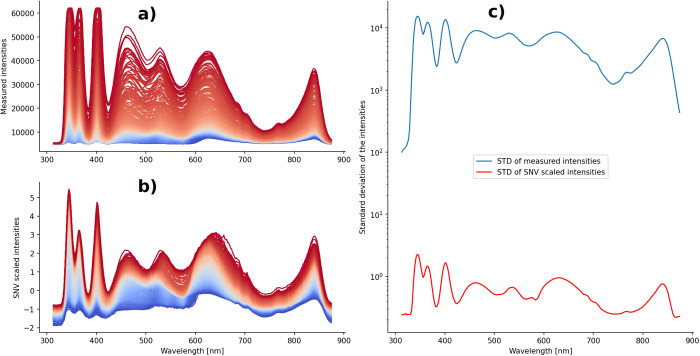
SNV correction on the spectral data measured in direct transmission with 200 µs exposure time (AT_200). **(a)** Original spectra. **(b)** Spectra after the SNV correction. **(c)** Semi-logarithmic plot of the standard deviation of the intensities before (blue line) and after (red line) the SNV correction.

The effect of the SNV method on our data is shown in Fig 3. In this example, all spectra measured in direct transmission with an exposure time of 200 µs (AT_200) are plotted together before (a) and after (b) the SNV correction. The effect of this scaling method on the variance of the data is shown in Fig 3. Here, for each wavelength, we show the standard deviation of the intensities of the spectra before (blue line) and after (red line) the SNV correction in a semi-logarithmic plot. With average values of 2517.71 (before SNV) and 0.19 (after SNV), the standard deviation of the intensities of each wavelength decreases drastically by 4 orders of magnitude. Similar results were obtained for all other spectrometer settings (defined by the combination of light pathway and exposure time).

#### Correlation analysis.

**Fig 4:**
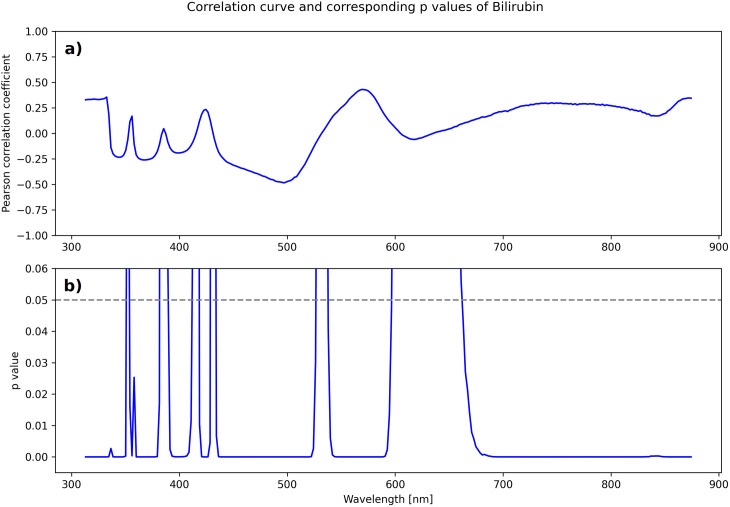
Correlation curve (a) and corresponding ρ-values (b) of Bilirubin. For the laboratory parameter “specific gravity” and spectrometer in direct transmission with 20 µs exposure time (DT_20). Only ρ-values smaller than 0.006 are shown in the plot.

In the second step of the evaluation, a correlation analysis was performed to identify the wavelengths in the spectrum that showed the strongest correlations with the measured laboratory parameters. The focus was placed on the most promising wavelengths, which were recognized as absorption maxima for specific parameters both in the analysis and in the literature. Regression models were then developed for these wavelengths (see Fig 4).

The measured spectra for a specific spectrometer setting can be arranged in an n× 288 matrix S where n represents the number of samples. Therefore, each column vector $s_{1}$ of the matrix contains all the scaled intensities measured at the i-th wavelength. Similarly, for a specific laboratory parameter, a vector $l=(l_{1},\;l_{2},...,l_{n})$ was defined to contain the measured laboratory values of each sample. A correlation coefficient and corresponding p-value were computed between each column vector $s_{1}$ and the laboratory vector l. This process was repeated for each spectrometer setting and laboratory parameter. Since all laboratory parameters were measured quantitatively, which is why Pearson’s correlation coefficients were computed.

#### Regression analysis.

To conduct a regression analysis for each parameter, wavelengths with sufficiently high and significant correlation coefficients (r> |0,3|; p < 0.01) were chosen from each light pathway. These selected wavelengths were then employed to generate a Tobit regression model [18] using R Statistical Software (v4.3.2; R Core Team 2023) and RStudio (v2023.09.1 + 494; RStudio Team 2023). For the analysis, the R packages ISLR [19], caret [20], glmtoolbox [21], dplyr [22], ConfusionTableR [23], tidyr [24], mlbench [25], mfp [26], olsrr [27], survival [28], censReg [29], and plm [30] were used for data import, cleaning, transformation, model building and evaluation, as well as for estimating specific regression models. This process included the following steps:

1. Dataset Splitting: To generate training and test data the dataset was randomly subdivided into five subsamples.
2. Fractional Polynomials: Multi-fractional polynomials were applied to the initial 80% of the data for transformation (training data).
3. Tobit Model and Backward Selection: The transformed data was integrated into a Tobit model, and backward selection was conducted to derive the final model.
4. Inner Sample Performance: The performance of the final model was assessed on the data used for training.
5. Out-of-Sample Performance: The performance of the final model was evaluated on the excluded data (remaining 20%, test data).
6. Performance Difference: The disparity between the inner sample performance and out-of-sample performance was calculated.

The steps described above were repeated five times, each time omitting a different subsample. Subsequently, the mean of the five differences was calculated. The steps 1–4 were then repeated for the entire dataset (MSE_global), and the mean was then subtracted from the inner sample performance of the global model (MSE_global_corrected). The regression analysis code, using hemoglobin as an example dataset from S4 Table, is presented in S1 and S2 File.

The advantage of this approach is to assess the robustness and general validity of the model by measuring its performance on both the training and test data, taking possible overfitting into account [31].

The tobit model was created both with and without considering random effects to capture the effect of repeated measurements that are not accounted for by the explanatory variables of the model. The best model was chosen to ensure the most accurate estimation of the parameters.

Finally, the Coefficient of Determination (R-squared) was computed. This metric indicates proportion of explained variance to total variance indicates that a higher $R^{2}$ value suggests that the model can explain a larger proportion of the variation in the data, indicating a better fit of the model to the data [32,33]. The formula is: $R^{2}=1-\frac{MSE_{Global-corrected}}{Varianz}$

#### Classification analysis.

Classification analyses were conducted in RStudio (v2023.09.1 + 494; RStudio Team 2023) to distinguish between pathological and non-pathological values. For each drainage parameter, a statistical model was created, which included both random effects (glmer) and no random effects (glm) [34]. Initially, datasets containing spectral data, drainage parameters, and anonymized patient identifications were randomized.

A threshold was set for each drainage parameter to distinguish pathological from non-pathological values. Subsequently, the pathological and non-pathological values were converted into a binary numerical coding (0 for normal value, 1 for pathological value). Spectral data were used as independent variables, while specific drainage parameters served as dependent variables. Each drainage parameter has been assigned a binary response variable. Additionally, wavelengths describing absorption maxima in the literature were included in the initial model whenever possible.

For the construction of classification models, the datasets were randomized and divided into five equally sized subsets (see Steps 1 and 2 in the regression analysis). The performance of the models was evaluated using metrics such as the Area Under the Curve (AUC) and the Balanced Accuracy (BAC) with the R packages caret [20], ConfusionTableR [23], and ROCR [35]. The glmtoolbox package [21] was employed to create initial generalized linear models (glm). Additionally, the lme4 package [34] was used to include random effects into the logistic regression models (glmer) concurrently. The validation of the global model was done by calculating the difference between the performance within and outside the sample. This mean was then subtracted from the performance within the sample to obtain the corrected AUC/BAC of the global model. The code for classification analysis, using the hemoglobin dataset from S4 Table as an example, is shown in S3 and S4 File.

**Fig 5:**
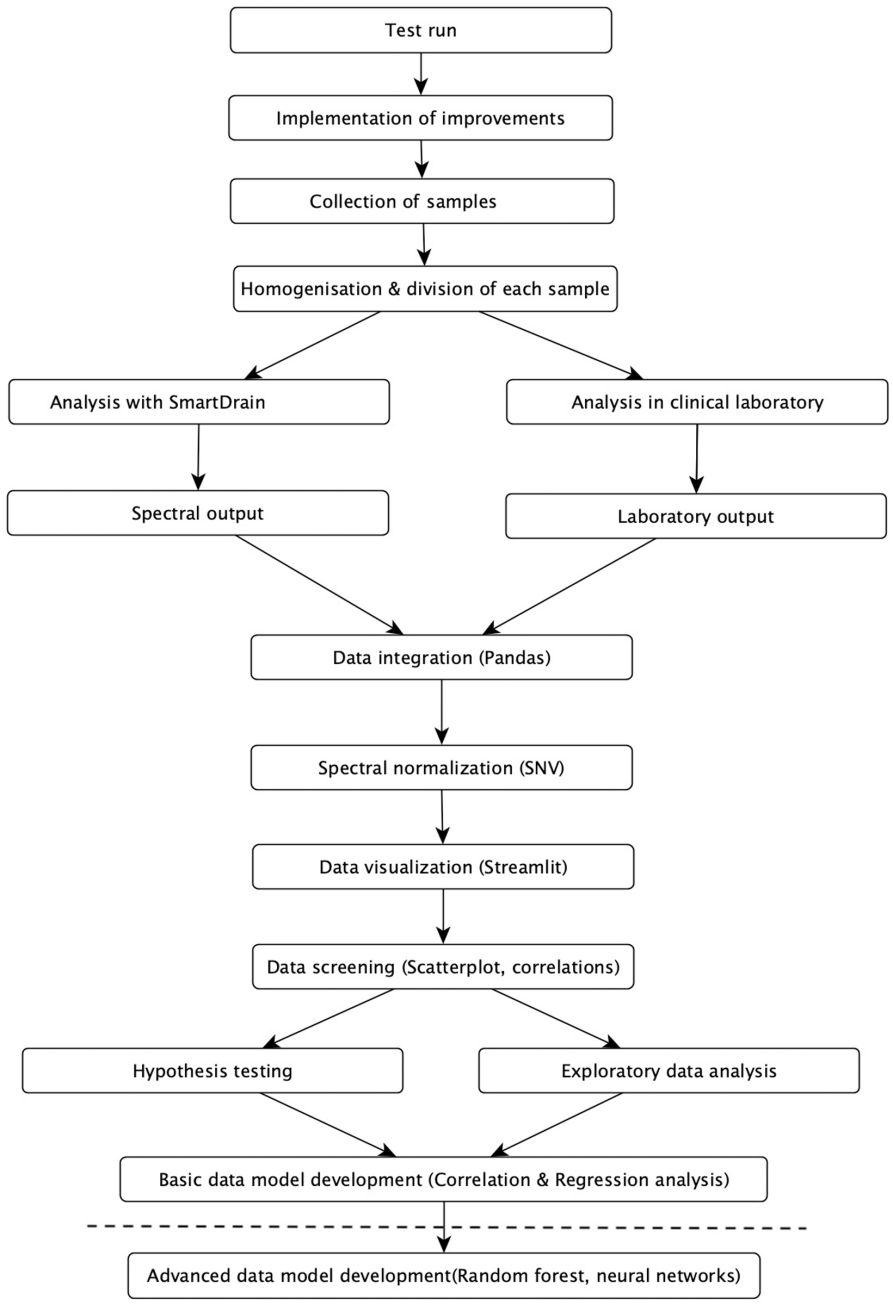
Flowchart of prototype development, data acquisition, data processing and statistical analysis.

Fig 5 provides an overview of the entire process of this study. The figure presents a visual representation of the key steps, emphasizing the flow and integration between each stage of the research process.

## Results

### Correlation analysis

The correlation analysis was conducted using correlation curves as the basis. All correlations discussed below have been deemed statistically significant, with a p-value of <0.01.

**Fig 6:**
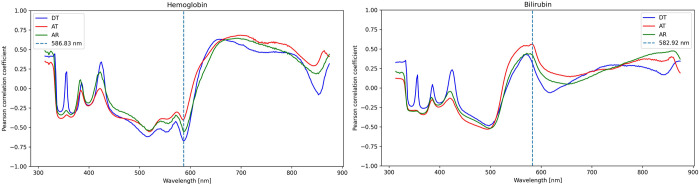
Correlation curves of hemoglobin and bilirubin. Pearson’s correlation coefficients on the x-axis and wavelength between 340 nm and 850 nm on the y-axis. DT (direct transmission) is the blue curve, AT (angular transmission) is red, and AR (angular reflection) is green.

Among these, the strongest correlations were observed for both hemoglobin (r = −0.67) and erythrocytes (r = −0.46) at a wavelength of 586 nm. Hemoglobin also exhibits notable correlations at 363 nm (r = −0.37), 514 nm (r = −0.62), and 557 nm (r = −0.45), suggesting the potential discernment of different hemoglobin forms (Fig 6). Additionally, erythrocytes show correlations at 518 nm (−0.425), 551 nm (r = −0.411), and 665 nm (r = 0.446). Bilirubin demonstrates absorption peaks at 582 nm (r = 0.56) and 496 nm (r = −0.49) (Fig 6).

Albumin and total protein showcase absorption peaks at 580 nm (r = −0.61) and 579 nm (r = −0.54), 682 nm (r = 0.58) and 672 nm (r = −0.56), and 532 nm (r = −0.56) and 537 nm (r = −0.49), respectively.

The strongest correlation for LDH is observed at 659 nm (r = 0.43), while uric acid exhibits its most prominent correlation at 354 nm with r = −0.4.

Triglycerides present absorption maxima at 588 nm and 667 nm with r > |0.34|. Conversely, lipase and amylase only show weak, significant correlations, with r < |0.3|.

No significant correlations were identified for leukocytes, mononuclear cells, polymorphonuclear cells, or glucose (see S1 Fig).

### Regression analysis

The wavelengths with statistically significant correlations were used for building regression models. Models incorporating a random effect exhibited superior performance, thus, models with random effects are presented herein.

For erythrocytes, the out-off-sample Mean Squared Error (MSE) stands at 0.0878. Our global model can account for 65.2% ($R^{2}$) of the variation in our dependent variable in the training data. Hemoglobin yields an MSE of 1.187, explaining around 63.9% of the training data. $R^{2}$≈ 0.529, suggesting the model can explain about 53% of the training data. Albumin exhibits an MSE of 33.74, with an R-squared value of 45.4% (see Table 3).

**Table 3:** Overview of MSE values of Tobit models with variance and R-squared.

Parameter/ MSEs	Erythrocytes	Hemoglobin	Bilirubin	Albumin	Total protein	Uric acid
**Tobit_1**	0.021	2.184	35.58	0.615	0.957	−4.399
**Tobit_2**	0.044	−0.698	−8.907	−0.371	− 0.133	−3.061
**Tobit_3**	−0.014	0.691	−14.132	−0.069	− 0.074	−7.006
**Tobit_4**	−0.029	−0.657	−20.432	0.316	− 0.608	7.211
**Tobit_5**	0.009	−1.101	11.140	−0.25	0.635	11.67
**MSE_global**	0.082	1.103	33.091	1.234	3.589	14.209
**MSE_global_corrected**	0.088	1.187	33.741	1.282	3.744	15.091
**Variance**	0.252	3.291	71.524	2.352	4.996	21.879
$R^{2}$	0.625	0.639	0.529	0.454	0.251	0.31

**Fig 7:**
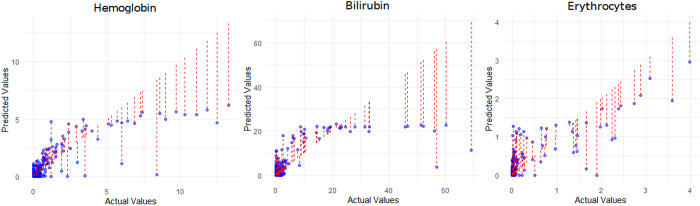
Scatter plots from the regression analysis of hemoglobin, erythrocytes, and bilirubin. In each plot, the actual values are shown on the X-axis and the predicted values on the Y-axis. Blue dots represent the predicted values for individual data points in comparison to the actual values. Red dashed lines indicate the deviation between the predicted and actual values for each point.

The models for uric acid and LDH possess a coefficient of determination R-squared of approximately 0.3. Models for lipase, amylase, and triglycerides were able to explain less than 10% of the variance in our dependent variable in the training data. Fig 7 displays scatter plots from the regression analysis of hemoglobin, erythrocytes, and bilirubin (scatter plots for all parameters are provided in S3 Fig). Each point represents a predicted value from the regressions model, while the red line indicates the deviation of the point from the actual value (from the central laboratory).

### Classification analysis

Classification analysis is illustrated using the example of hemoglobin. The final logistic regression model for hemoglobin see in Table 4 (Models for all parameters are provided in S1 Table).

**Table 4:** Final logistic regression model with (glmer) and without random intercept (glm) for hemoglobin with covariates wavelength.

Parameter	Model	Covariates
Hemoglobin	glmer	DT_342.41nm, AT_363.92nm, AR_363.92nm, AR_557.5nm, DT_EX1_586.83nm
	glm	DT_342.41nm, AT_363.92nm, AR_363.92nm, AR_557.5nm, DT_586.83nm

The performance of the logistic regression model with random intercept was evaluated both within and outside the sample. In the “Inner sample” analysis, the AUC ranged between 0.98 and 0.975, while the BAC ranged from 0.93 to 0.907. The global model attained an AUC of 0.977 and a global BAC of 0.905.

Regarding the analysis conducted out-of-sample performance, the AUC ranged from 0.97 to 0.917, and the BAC ranged from 0.906 to 0.838. The mean differentials between inner and out-of-sample performance for the logistic regression model with random intercept were calculated 0.03 (AUC), with a variance of 0.0006 and a standard deviation of 0.024. Similarly, the mean differentials between inner and out-of-sample performance for BAC was 0.11, with a variance of 0.005 and a standard deviation of 0.07. The global corrected model demonstrated an AUC of 0.947 (Table 5) and a BAC of 0.853.

**Fig 8:**
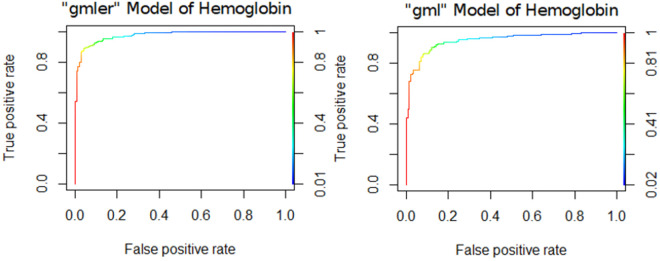
ROC curves for “gmler” and “gml” models of hemoglobin. X-axis: false positive rate, Y-axis: true positive rate.

The performance of the logistic regression model without random intercept in the inner sample analysis showed an area under the curve (AUC) ranging from 0.895 to 0.877, with a balanced accuracy (BAC) between 0.956 and 0.9475. Conversely, in the out-of-sample analysis, the AUC varied between 0.959 and 0.924, and the BAC ranged from 0.92 to 0.858.On a global scale, the model achieved an AUC of 0.95 and a BAC of 0.891. Furthermore, the mean differentials between inner and out-of-sample performance for AUC across iterations was 0.005, with a standard deviation (SD) of 0.019 and a variance of 0.00037. The global corrected model yielded an AUC of 0.945 (Fig 8). S4 Fig shows the ROC curves for the ‘gmler’ and ‘gml’ models of all parameters.

Similarly, the mean differentials between inner and out-of-sample performance for BAC, the value was 0.001 with an SD of 0.032 and a variance of 0.001. The global corrected BAC model was 0.889.The mean differentials of the models for all parameters are provided in S2 Table.

In Table 5, models with and without random effects (+RE/-RE) for all parameters are presented.

**Table 5:** Overview of the classification analysis models for all drainage parameters.

Parameter\model	AUC + RE	BAC + RE	AUC-RE	BAC-RE
**Hemoglobin**	0.947	0.853	0.945	0.889
**Triglycerides**	0.941	0.789	0.827	0.778
**LDH**	0.94	0.806	0.893	0.757
**Bilirubin**	0.918	0.807	0.723	0.722
**Erythrocytes**	0.905	0.792	0.897	0.740
**Total Protein**	0.884	0.914	0.797	0.725
**Albumin**	0.882	0.741	0.877	0.757
**Uric acid**	0.856	0.831	0.746	0.613
**Amylase**	0.845	0.762	0.702	0.526
**Lipase**	0.826	0.75	0.666	0.582

## Discussion

In 2017, van Duren et al. described a digital technology to measure volume, making use of a positive terminal at the lowest point of the vessel and negative (sensor) terminals placed at accurate intervals along an axis of the vessel. A few years ago, there was an approach providing a solution for digital measurement of drain volume [13]. Although a comprehensive usage of such a technique in daily clinical practice would significantly improve drain management, the disadvantages of a manual analysis of the output quality would remain.

This study examined the light absorption properties of drainage fluids using a compact mini-spectrometer. The ultimate goal is to facilitate the digitalization of drainage fluid analysis and to enhance postoperative drain management and patient care.

Our correlation analysis revealed a linear relationship between certain parameters at different wavelengths. Values between 0,5 and 0,7 are described as moderately strong positive or negative linear correlations [36]. Accordingly, hemoglobin, bilirubin, albumin, and total protein show a moderately strong correlation at specific wavelengths. Correlation coefficients between 0,3 and 0,5 indicate a weak positive or negative linear correlation, as observed for erythrocytes, uric acid, LDH, triglycerides, and glucose. Weak linear relationships were observed for parameters such as amylase and lipase only. Cells such as leukocytes, mononuclear cells, and polymorphonuclear cells, which do not absorb in the visible light spectrum, show no or minimal correlations and poor discriminatory ability in classification and regression models. The wavelengths corresponding to the absorption maxima of hemoglobin [37], erythrocytes, and bilirubin [38] align with the measurement ranges documented in the literature. Additionally, they resemble the findings of the pilot study, highlighting the consistency and reproducibility of our results [14].

The analysis of MSE values and variance from the Tobit models showed that reliable prediction models can be created for erythrocytes, hemoglobin, bilirubin, and albumin. However, the models for total protein and uric acid are slightly less accurate. Our findings demonstrate that the actual concentrations of hemoglobin and bilirubin can be estimated using spectral analysis. For hemoglobin, the regression model, with an R^2^ value of 0.639, accounted for 64% of the variation in the training data, reflecting relatively high accuracy.

The Mean Squared Error (MSE) of 1.187 further supports the reliability of the predictions.

In contrast, the model for bilirubin showed a moderate correlation, with an R^2^ value of 0.529 and an MSE of 33.741, indicating somewhat less reliable predictions.

Based on the correlation analysis at known wavelengths, good classification models were developed to distinguish between pathological and non-pathological values. The classification guidelines are as follows: values below 0.6 indicate poor discrimination, between 0.7 and 0.8 range from poor to good, above 0.8 are considered good, and above 0.9 are very good [39]. Using these criteria, our models showed very good discrimination for hemoglobin, triglycerides, LDH, bilirubin, and erythrocytes in at least one model, while total protein, albumin, uric acid, amylase, and lipase showed good discrimination. The ROC curves for these models ranged from good to very good. Both the classification and regression analyses revealed that models considering random effects produce better results.

Our study has some limitations. First, the patient cohort might not be representative of the broader population, limiting the generalizability of the findings. To counter this, we selected a diverse and sufficiently large sample of patients. Additionally, compact mini-spectrometers may have lower sensitivity and accuracy compared to larger, more sophisticated instruments, potentially affecting the reliability of the measurements. We performed rigorous calibration and validation against larger instruments to minimize this possibility.

Surgical drain fluids can vary widely in composition based on factors such as the type of surgery, patient health, and even individual recovery processes, introducing significant variability. This variability may impact the generalizability of the study’s findings, as the models developed in this study may perform differently when applied to other patient populations or clinical settings. To mitigate this, we made it a point to analyse all samples whenever possible, independent of their viscosity and color. However, the diverse nature of surgical drain fluid composition means that further studies are needed to assess how this variability could affect the performance and applicability of the models in different clinical scenarios.

Additionally, inconsistencies in fluid collection, storage, and handling could introduce variability and affect the integrity of the samples and the subsequent spectral analysis. We minimized this through standardized protocols and training for all personnel involved. Similarly, external factors such as light, temperature, and humidity can influence spectrometer readings, potentially leading to inaccuracies. All of these variables were controlled by conducting the measurements in a controlled environment. The accuracy of the spectrometer data depends on the proper calibration and operation of the device, requiring significant technical expertise. Accuracy was assured by comprehensive calibration of the spectrometers.

Lastly, spectral data can be affected by interference and noise, which may obscure subtle differences in fluid composition. We reduced this issue by employing advanced data processing and noise reduction techniques. Some parameters, such as glucose, could not be detected with the currently used spectrum because glucose’s absorption spectrum lies in the infrared range beyond 780 nm [40]. To address this gap and obtain more comprehensive information, we plan to extend the spectrometer’s measurement range in the future. This extension will allow us to detect substances whose absorption spectra are currently outside the existing measurement range. By making this adjustment, we aim to enhance the precision of our analyses and provide valuable additional data for clinical applications.

The results of the regression analysis suggest that spectral analysis could be used as a supportive tool in clinical practice. However, the accuracy of the parameters is not yet sufficient to recommend this method as the sole basis for determining exact concentrations in a routine clinical setting. Our study suggests that this method still needs to be further refined before it can be fully integrated into clinical practice to precisely determine the exact concentrations of biomarkers such as hemoglobin and bilirubin.

Despite these limitations, it is worthwhile to consider the outlook of the approach described here. The use of our smart drains in daily clinical practice could result in a tremendous improvement in patient care. Continuous real-time monitoring of crucial clinical parameters can detect even small changes in the surgical drain output during the recovery process, potentially identifying complications such as bleeding, infection, or leakage early and without the reliance on additional personnel.

The current evaluation platform represents a comprehensive investigation in which multiple illumination angles are examined to optimize diagnostic accuracy. Based on these extensive studies, only the most effective measurement angle will be incorporated in the final product, resulting in a compact (3 × 3 × 6 cm) wireless diagnostic device specifically designed for drainage monitoring. Advanced statistical models and AI algorithms are currently being implemented to enhance parameter analysis. Promising improvements in AUC values (Area Under the Curve) and Balanced Accuracy (BAC) have been demonstrated in preliminary analyses, establishing the foundation for an AI-powered system capable of early complication detection and prediction of hospital length of stay.

To further validate and expand the clinical utility of the Smart Drain system, several important research directions must be pursued. Primary focus is being placed on the development of true real-time analysis capabilities at the bedside. This advancement would eliminate current delays associated with laboratory analysis and significantly improve postoperative monitoring. Additionally, the biomarker panel is being expanded to include detection of lipase and amylase in drainage fluid, which would be particularly valuable for pancreatic and abdominal surgeries, where early detection of anastomotic leaks or pancreatitis is considered critical.

In the next research phase, multicenter validation studies will be conducted with diverse surgical patient groups to rigorously assess the system’s generalizability. The capability for continuous real-time biomarker monitoring could potentially transform patient management across multiple surgical specialties, particularly in high-risk procedures and complex medical cases. Particular potential has been demonstrated for early detection of critical complications such as bleeding, infections, and organ dysfunction across various surgical disciplines—ranging from cardiothoracic and gastrointestinal to orthopedic and neurosurgical procedures. Beyond surgical drains, adaptations of this technology are being explored for other clinically relevant bodily fluids including ascites, pleural effusions, cerebrospinal fluid, and urine, potentially opening new diagnostic possibilities in intensive care and internal medicine. [41]

Moving forward, efforts are being made to integrate AI-based diagnostic algorithms into clinical routines to improve monitoring precision, optimize patient outcomes, and refine postoperative care pathways. As this technology continues to be developed through rigorous clinical validation, it is anticipated to become an essential surgical tool that enables both personalized patient management and generates valuable data for broader clinical applications. The compact, wireless design of the final product is expected to facilitate seamless integration into existing clinical environments and potentially establish a new standard for postoperative monitoring.

In conclusion, the Smart Drain system demonstrates transformative potential for postoperative care through its innovative real-time monitoring capabilities. As clinical validation continues, this technology is poised to establish new standards for complication detection and patient management in surgical settings. The system’s compact design and AI-enhanced analytics position it as a promising tool for improving outcomes and optimizing healthcare resource utilization.

## Supporting information

S1 Table Final logistic models of drain parameters. “gmler” with random effect; “gml” without random effect.
(PDF)

S2 Table Mean differences of the models BAC (Balance Accuracy) and AUC (Area Under the Curve) with and without random effect (+/-RE).
(PDF)

S3 Table Laboratory values and spectral data. The table contains all laboratory values in columns F–S. Column A lists the randomized patient numbers. Columns C–E contain the spectral data corresponding to each sample and each exposure setting (DT, AT, AR).
(XLSX)

S4 Table Hemoglobin input dataset for analysis in R Studio. Column A: Binary classification of data based on laboratory values from Column B (cut-off value = 0: 0 = 0, > 0 = 1); Column B: Laboratory values; Column C: Randomized patient numbers; Columns E–H: Wavelengths with corresponding spectral data.
(XLSX)

S1 Fig Correlations curves of drain parameters. Pearson’s correlation coefficients on the x-axis and wavelength between 340 nm and 850 nm on the y-axis. DT (direct transmission) is the blue curve, AT (angular transmission) is red, and AR (angular reflection) is green. On the X-axis, a wavelength is selected, with its correlation coefficient marked on the curve.
(TIF)

S2 Fig Scatter plots of drainage parameters. Specific wavelengths and illumination angles: DT (direct transmission) is blue, AT (angular transmission) is red.
(TIF)

S3 Fig Scatter plots from the regression analysis of drainage parameters. Each point represents a predicted value from the regressions model, while the red line indicates the deviation of the point from the actual value (from the central laboratory).
(TIF)

S4 Fig ROC curves for logistic regressions models with random intercept of drainage parameters. X-axis: false positive rate, Y-axis: true positive rate.
(TIF)

S1 File Code for a regression Tobit model with random effects in R as an example for hemoglobin (partly in German). (PDF)

S2 File Code for regression tobit model without random effects in R as an example for hemoglobin (partly german). (PDF)

S3 File Code for classifikation with random effects as an example for hemoglobin (partly german). (PDF)

S4 File Code for classifikation without random effects as an example for hemoglobin (partly german). (PDF)
