# Supplementary material for: Intelligent surgical drainage - digitizing the analysis of drainage fluid in patients with surgical drains
Source: PLoS One. 2025 Jul 28;20(7):e0325072. doi: 10.1371/journal.pone.0325072 (PMC12303269; doi:10.1371/journal.pone.0325072)
Supplement: S1 Table — Final logistic models of drain parameters. “gmler” with random effect; “gml” without random effect. (PDF) [file pone.0325072.s001.pdf]

Parameter	Model	Covariates
Hemoglobin	glmer	DT 342.41nm, AT 363.92nm, AR 363.92nm, AR 557.5nm, DT EX1 586.83nm
	glm	DT 342.41nm, AT 363.92nm, AR 363.92nm, AR 557.5nm, DT 586.83nm
Triglycerides	glmer	DT 514.48nm, DT 588.79nm, DT 667.0nm
	glm	DT 514.48nm, DT 588.79nm, DT 667.0nm
LDH	glmer	AR 344.37nm, AR 363.92nm, AR 440.18nm, AR 555.55nm, AR 582.92nm, AT 659.18nm
	glm	AR 344.37nm, AR 363.92nm, AR 440.18nm, AR 555.55nm, AR 582.92nm, AT 659.18nm
Bilirubin	glmer	DT 496.88nm, AT 582.92nm, DT 745.22nm
	glm	DT 496.88nm, AT EX2 582.92nm, DT 745.22nm
Erythrocytes	glmer	DT 518.39nm, DT 551.63nm, DT 586.83nm, DT 655.27nm
	glm	DT 518.39nm, DT 551.63nm, DT 586.83nm, DT 655.27nm
Total Protein	glmer	DT 344.37nm, DT 401.07nm, AT 442.13nm, AT 537.95nm, AT 672.87nm
	glm	DT 344.37nm, DT 401.07nm, AT 442.13nm, AT 537.95nm, AT 672.87nm
Albumin	glmer	DT 346.32nm, DT 401.07nm, AT 448.0nm, AT 532.08nm, AT 682.64nm
	glm	DT 346.32nm, DT 401.07nm, AT 448.0nm, AT 532.08nm, AT 682.64nm
Uric acid	glmer	DT 334.59nm, DT 354.14nm, AT 403.03nm, DT 434.31nm, AR 444.09nm
	glm	DT 334.59nm, DT 354.14nm, AT 403.03nm, DT 434.31nm, AR 444.09nm
Amylase	glmer	DT 383.47nm, AT 532.08nm, DT 571.19nm, DT 610.3nm
	glm	DT 383.47nm, AT 532.08nm, DT 571.19nm, DT 610.3nm
Lipase	glmer	DT 334.59nm+DT 383.47nm, AT 537.95nm+AT 575.1nm, AR 625.94nm
	glm	DT 334.59nm, DT 383.47nm, AT 537.95nm, AT 575.1nm, AR 625.94nm
