# Supplementary material for: Intelligent surgical drainage - digitizing the analysis of drainage fluid in patients with surgical drains
Source: PLoS One. 2025 Jul 28;20(7):e0325072. doi: 10.1371/journal.pone.0325072 (PMC12303269; doi:10.1371/journal.pone.0325072)
Supplement: S2 Table — Mean differences of the models BAC (Balance Accuracy) and AUC (Area Under the Curve) with and without random effect (+/-RE). (PDF) [file pone.0325072.s002.pdf]

**Mean differences/Parameter**	**Mean BAC+RE**	**Mean BAC-RE**	**Mean AUC+RE**	**Mean AUC-RE**
Hemoglobin	0.1143801	0.001328943	0.02949041	0.005396363
Triglycerides	0.0907102	-0.07202518	0.05635067	-0.002353355
LDH	0.1154546	-0.002269696	0.0977611	0.009175642
Bilirubin	0.1143801	0.003329903	0.072289	0.002577754
Erythrocytes	0.03921166	0.01136019	0.045217	0.009614387
Total Protein	0.09994911	-0,00003	0.08466392	0.00097
Albumin	0.1674038	-0,00009	0.1082982	0.002375752
Uric acid	0.09745377	0.002993713	0.1402891	0.03195366
Amylase	0.1246804	0.01752333	0.1357311	0.03953832
Lipase	0.15716	0.008801347	0.1547557	0.03899263
