# Supplementary figures and images for: Intelligent surgical drainage - digitizing the analysis of drainage fluid in patients with surgical drains
Source: PLoS One. 2025 Jul 28;20(7):e0325072. doi: 10.1371/journal.pone.0325072 (PMC12303269; doi:10.1371/journal.pone.0325072)

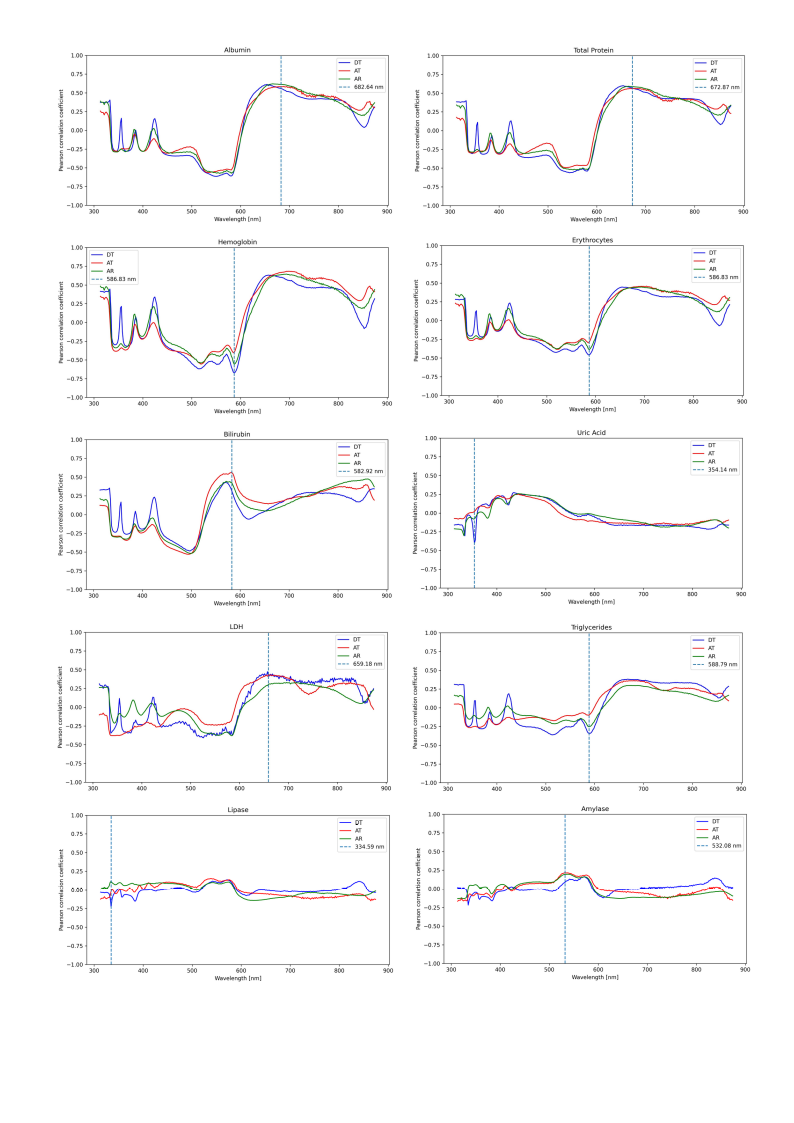

Supplement: S1 Fig — Pearson’s correlation coefficients on the x-axis and wavelength between 340 nm and 850 nm on the y-axis. DT (direct transmission) is the blue curve, AT (angular transmission) is red, and AR (angular reflection) is green. On the X-axis, a wavelength is selected, with its correlation coefficient marked on the curve. (TIF) [file pone.0325072.s005.tif]

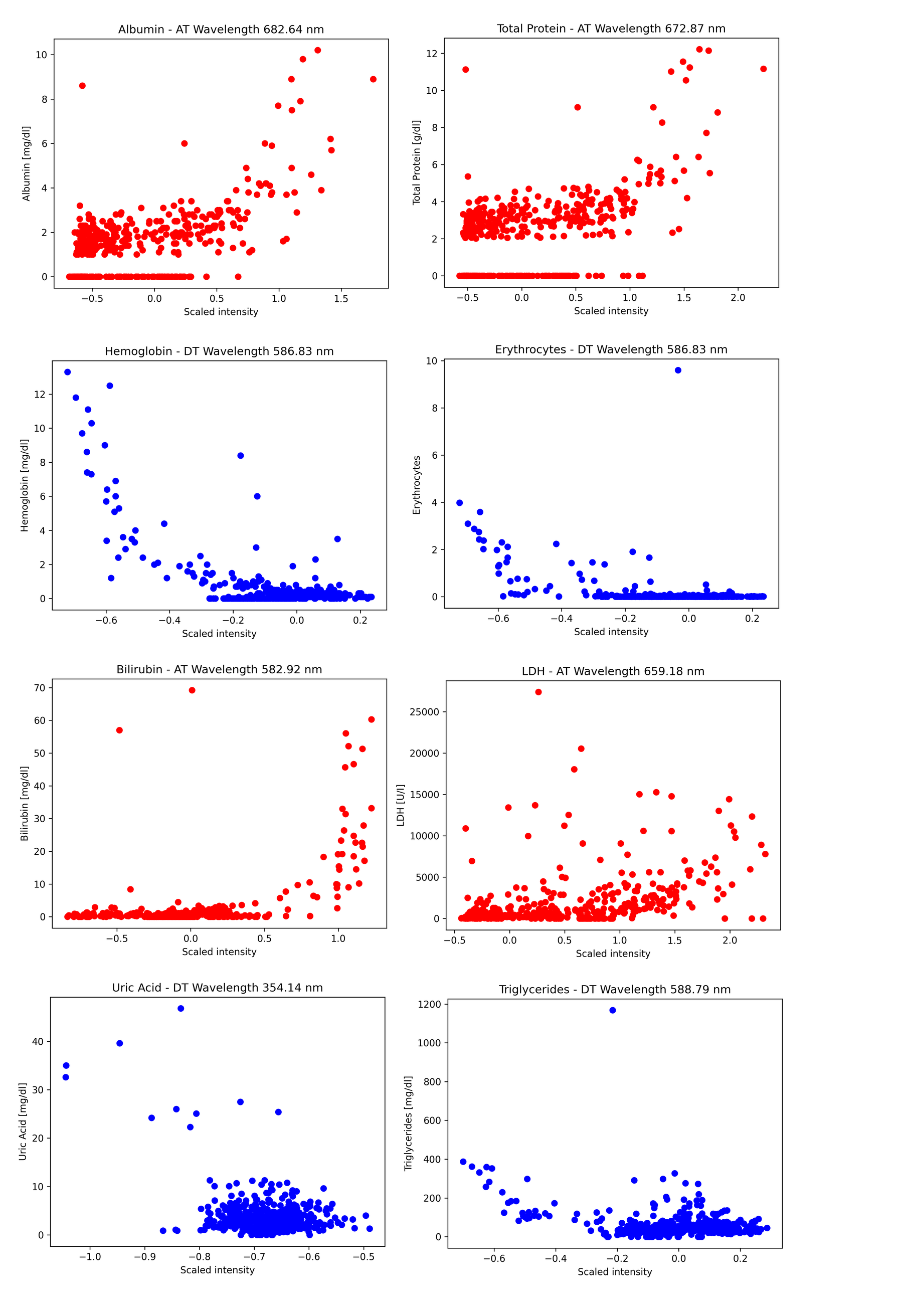

Supplement: S2 Fig — Specific wavelengths and illumination angles: DT (direct transmission) is blue, AT (angular transmission) is red. (TIF) [file pone.0325072.s006.tif]

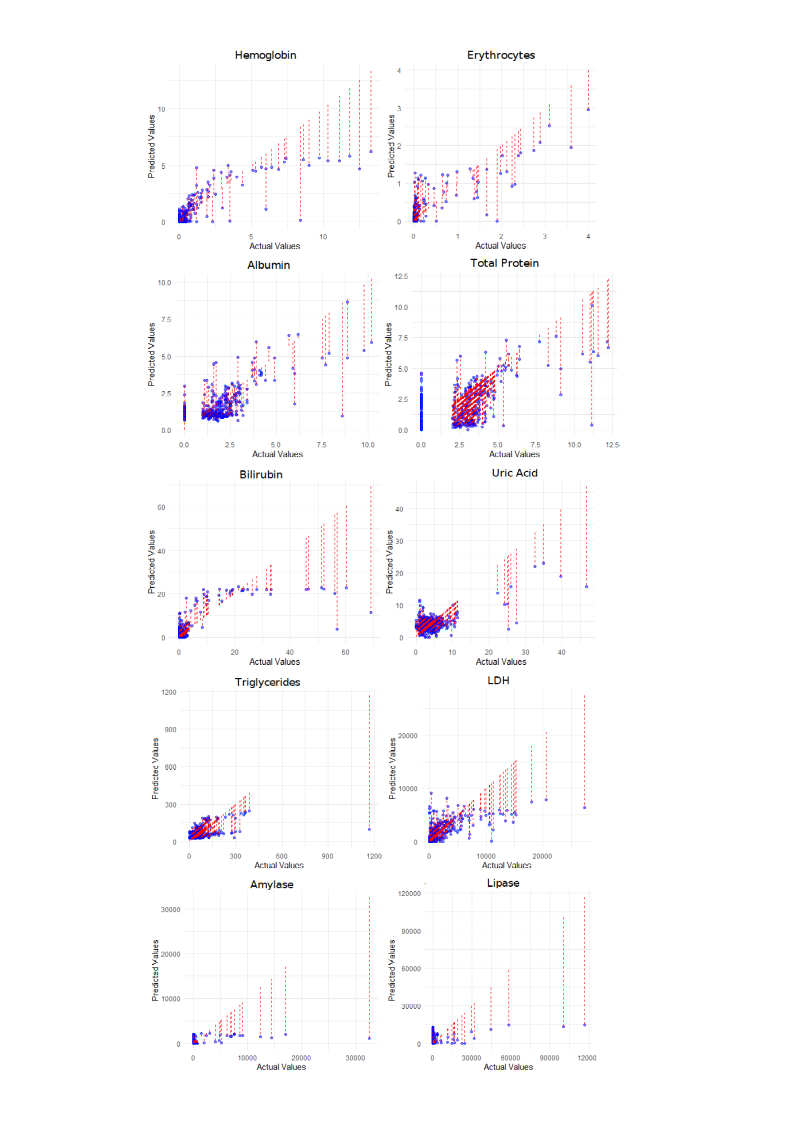

Supplement: S3 Fig — Each point represents a predicted value from the regressions model, while the red line indicates the deviation of the point from the actual value (from the central laboratory). (TIF) [file pone.0325072.s007.tif]

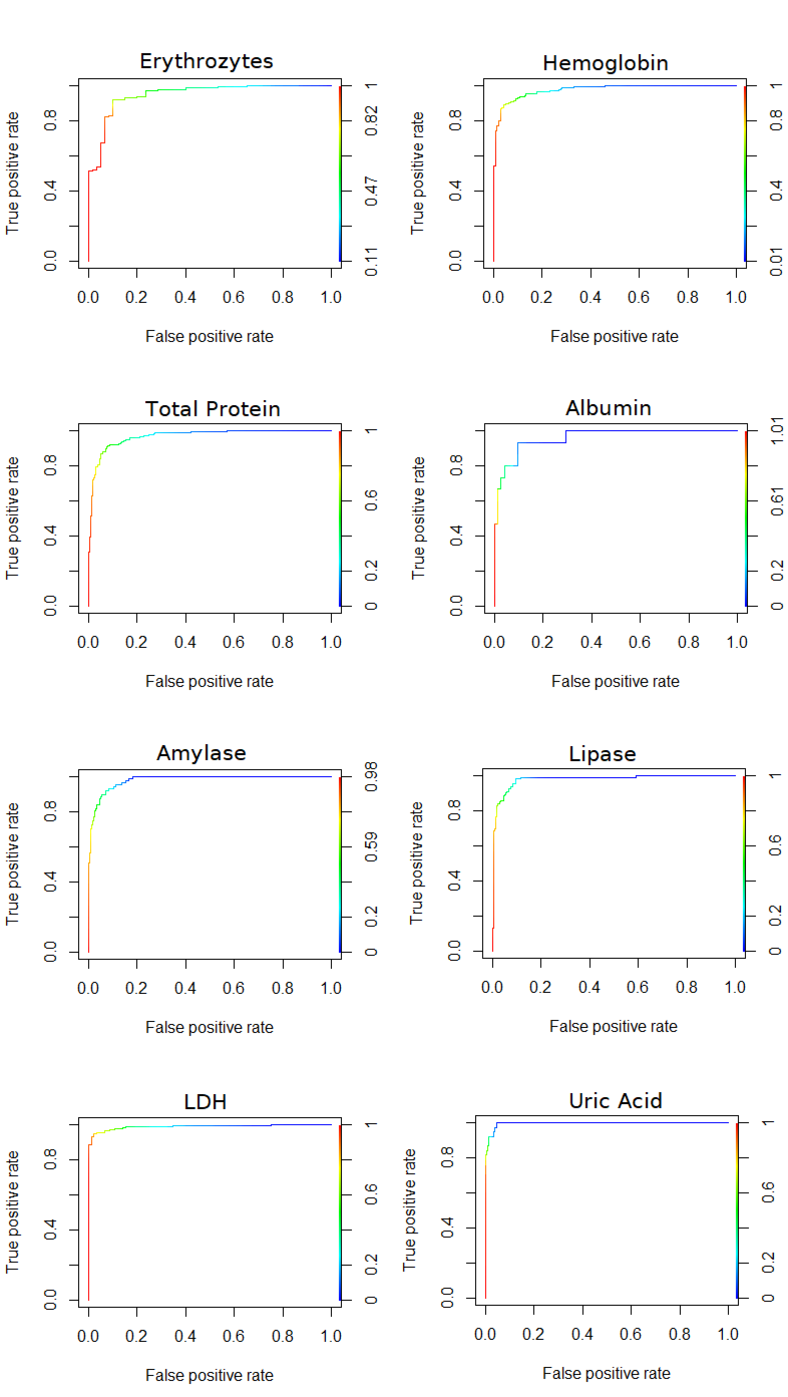

Supplement: S4 Fig — X-axis: false positive rate, Y-axis: true positive rate. (TIF) [file pone.0325072.s008.tif]
