# Supplementary material for: Intelligent surgical drainage - digitizing the analysis of drainage fluid in patients with surgical drains
Source: PLoS One. 2025 Jul 28;20(7):e0325072. doi: 10.1371/journal.pone.0325072 (PMC12303269; doi:10.1371/journal.pone.0325072)
Supplement: S1 File — (PDF) [file pone.0325072.s009.pdf]

```
#install.packages("ISLR")
library(ISLR)
#install.packages("caret")
library(caret)
#install.packages("glmtoolbox")
library(glmtoolbox)
#install.packages("dplyr")
library(dplyr)
#install.packages("ConfusionTableR")
library(ConfusionTableR)
#install.packages("tidyr")
library(tidyr)
#install.packages("mlbench")
library(mlbench)
#install.packages("mfp") #wird fÃ¼r fractional polynomial
benÃ¶tigt
library(mfp)
#working directory
setwd("F:/1. Klinisches Semester/Promotion/R Analyse")
#Daten werden eingelesen
#install.packages("readxl")
library(readxl)
#install.packages("olsrr")     #wird fÃ¼r die model selection
benÃ¶tigt
library(olsrr)
#install.packages("survival")
library(survival)
#install.packages("censReg")
library(censReg)
#install.packages("plm") #wird für die Berücksichtigung von
Random Effects benötigt
library(plm)
```

```
#################################################################
######

Hämoglobin <- read_excel("7_hemoglobin input dataset for R.xlsx")

Hämoglobin_Random_Effects<-subset(Hämoglobin,
select=c("Hämoglobin", "DT_EX1_342.41nm", "AT_EX2_363.92nm",
"AR_EX2_363.92nm", "AR_EX2_557.5nm", "DT_EX1_586.83nm",

"Patient"))


Hämoglobin<-Hämoglobin_Random_Effects

#Hämoglobin als panel data frame definieren, um bei censReg
Random Effects zu berücksichtigen

#Hämoglobin<- pdata.frame( Hämoglobin, index = "Patient")

####################### Minima der Regressoren
####################

hist(Hämoglobin$DT_EX1_342.41nm)

hist(Hämoglobin$AT_EX2_363.92nm)

hist(Hämoglobin$AR_EX2_363.92nm)

hist(Hämoglobin$AR_EX2_557.5nm)

hist(Hämoglobin$DT_EX1_586.83nm)


min (Hämoglobin$DT_EX1_342.41nm)

#-0.9256574

min(Hämoglobin$AT_EX2_363.92nm)

#-1.054466

min(Hämoglobin$AR_EX2_363.92nm)

#-1.011524

min(Hämoglobin$AR_EX2_557.5nm)

#-0.9856557

min(Hämoglobin$DT_EX1_586.83nm)

#-0.7216694
```

```
##################################################################
##################################################################
###################################

### Einführung einer künstlichen unteren Grenze der Regressoren
###

Hämoglobin$DT_EX1_342.41nm <- ifelse(Hämoglobin$DT_EX1_342.41nm
< -2, -2, Hämoglobin$DT_EX1_342.41nm)

Hämoglobin$AT_EX2_363.92nm <- ifelse(Hämoglobin$AT_EX2_363.92nm
< -2, -2, Hämoglobin$AT_EX2_363.92nm)

Hämoglobin$AR_EX2_363.92nm <- ifelse(Hämoglobin$AR_EX2_363.92nm
< -2, -2, Hämoglobin$AR_EX2_363.92nm)

Hämoglobin$AR_EX2_557.5nm <- ifelse(Hämoglobin$AR_EX2_557.5nm <
-2, -2, Hämoglobin$AR_EX2_557.5nm)

Hämoglobin$DT_EX1_586.83nm <- ifelse(Hämoglobin$DT_EX1_586.83nm
< -2, -2, Hämoglobin$DT_EX1_586.83nm)


#Addieren einer Konstanten, damit keine negativen Regressorwerte
im Testdatensatz auftauchen

Hämoglobin$DT_EX1_342.41nm<-Hämoglobin$DT_EX1_342.41nm+2.1

Hämoglobin$AT_EX2_363.92nm<-Hämoglobin$AT_EX2_363.92nm+2.1

Hämoglobin$AR_EX2_363.92nm<-Hämoglobin$AR_EX2_363.92nm+2.1

Hämoglobin$AR_EX2_557.5nm<-Hämoglobin$AR_EX2_557.5nm+2.1

Hämoglobin$DT_EX1_586.83nm<-Hämoglobin$DT_EX1_586.83nm+2.1


#################################################

### Aufteilen des Datensatzes in 5 Teilmengen ###

#################################################

set.seed(2023)

sample_size <- nrow(Hämoglobin)

set_proportions <- c(Gruppe1 = 0.2, Gruppe2 = 0.2, Gruppe3 = 0.2,
Gruppe4= 0.2, Gruppe5=0.2)

set_frequencies <- diff(floor(sample_size * cumsum(c(0,
set_proportions))))

Hämoglobin$set <- sample(rep(names(set_proportions), times =
set_frequencies))

#Erstellen der 5 subsamples
```

```
Gruppe1 <- Hämoglobin[Hämoglobin$set == "Gruppe1", ]
Gruppe2 <- Hämoglobin[Hämoglobin$set == "Gruppe2", ]
Gruppe3 <- Hämoglobin[Hämoglobin$set == "Gruppe3", ]
Gruppe4 <- Hämoglobin[Hämoglobin$set == "Gruppe4", ]
Gruppe5 <- Hämoglobin[Hämoglobin$set == "Gruppe5", ]

#Definiere die 5 DatensÃ¤tze, an denen die Schritte 3
durchgefÃ¼hrt werden
Datensatz_4_1 <- rbind(Gruppe2, Gruppe3, Gruppe4, Gruppe5)
Datensatz_4_2 <- rbind(Gruppe1, Gruppe3, Gruppe4, Gruppe5)
Datensatz_4_3 <- rbind(Gruppe1, Gruppe2, Gruppe4, Gruppe5)
Datensatz_4_4 <- rbind(Gruppe1, Gruppe2, Gruppe3, Gruppe5)
Datensatz_4_5 <- rbind(Gruppe1, Gruppe2, Gruppe3, Gruppe4)

#Datensätze als panel data frame definieren, um bei censReg
Random Effects zu berücksichtigen
Datensatz_4_1<- pdata.frame( Datensatz_4_1, index = "Patient")
Datensatz_4_2<- pdata.frame( Datensatz_4_2, index = "Patient")
Datensatz_4_3<- pdata.frame( Datensatz_4_3, index = "Patient")
Datensatz_4_4<- pdata.frame( Datensatz_4_4, index = "Patient")
Datensatz_4_5<- pdata.frame( Datensatz_4_5, index = "Patient")

Gruppe1<- pdata.frame( Gruppe1, index = "Patient")
Gruppe2<- pdata.frame( Gruppe2, index = "Patient")
Gruppe3<- pdata.frame( Gruppe3, index = "Patient")
Gruppe4<- pdata.frame( Gruppe4, index = "Patient")
Gruppe5<- pdata.frame( Gruppe5, index = "Patient")



Hämoglobin<- pdata.frame( Hämoglobin, index = "Patient")
###################
### Datensatz 1 ###
```

```
###################


### fractional polynomials 1 ###

#df=4 !

model_fractional_1<- mfp(Hämoglobin ~fp( DT_EX1_342.41nm, df = 4, select = 0.05) + fp( AT_EX2_363.92nm, df = 4, select = 0.05) + fp( AR_EX2_363.92nm, df = 4,

                                  select = 0.05)+
fp( AR_EX2_557.5nm, df = 4, select = 0.05) +
fp( DT_EX1_586.83nm, df = 4, select = 0.05) , data=Datensatz_4_1)

model_fractional_1


#Datentransformation 1

#Trainingsdaten

Datensatz_4_1$AT_EX2_363.92nm_new<-I((Datensatz_4_1$AT_EX2_363.92nm/10)^1)

Datensatz_4_1$AR_EX2_557.5nm_new<-I(Datensatz_4_1$AR_EX2_557.5nm^1)

Datensatz_4_1$DT_EX1_586.83nm_new<-I(Datensatz_4_1$DT_EX1_586.83nm^-2)+I(Datensatz_4_1$DT_EX1_586.83nm^-2*log(Datensatz_4_1$DT_EX1_586.83nm))


#Testdaten

Gruppe1$AT_EX2_363.92nm_new<-I((Gruppe1$AT_EX2_363.92nm/10)^1)

Gruppe1$AR_EX2_557.5nm_new<-I(Gruppe1$AR_EX2_557.5nm^1)

Gruppe1$DT_EX1_586.83nm_new<-I(Gruppe1$DT_EX1_586.83nm^-2)+I(Gruppe1$DT_EX1_586.83nm^-2*log(Gruppe1$DT_EX1_586.83nm))


#Tobit Modell für Datensatz_4_1 mit Random Effects

tobit_1<-censReg(Hämoglobin ~ AT_EX2_363.92nm_new + AR_EX2_557.5nm_new + DT_EX1_586.83nm_new, left = 0,

                 right = Inf, data = Datensatz_4_1, method = "BHHH")


#backward selection
```

```
step(tobit_1,direction="backward") #AR_EX2_557.5nm_new  wird
entfernt


summary(tobit_1)




tobit_1<-censReg(Hämoglobin ~ AT_EX2_363.92nm_new +
DT_EX1_586.83nm_new , left = 0,

                 right = Inf, data = Datensatz_4_1 , method =
"BHHH" )

summary(tobit_1)




#Geschätzte Koeffizienten als Schätzer definieren

Schätzer1<-tobit_1$estimate

Schätzer1

Covariates.1 <-
data.frame(1,Datensatz_4_1[,names(Schätzer1[1:(length(Schätzer1)
-2)])[2:(length(Schätzer1)-2)]])

Covariates.1

Coefficients.1 <- Schätzer1[1:(length(Schätzer1)-2)]

Coefficients.1

head(as.matrix(Covariates.1) %*% as.matrix(Coefficients.1))

#predicted values

Datensatz_4_1$prediction_inner <- as.matrix(Covariates.1) %*%
as.matrix(Coefficients.1)

Datensatz_4_1$prediction_inner

Datensatz_4_1$prediction_inner <-
ifelse(Datensatz_4_1$prediction_inner < 0, 0,
Datensatz_4_1$prediction_inner)

Datensatz_4_1$prediction_inner


### MSE berechnen 1###

MSE_DF_1.1 <- data.frame(pred=Datensatz_4_1$prediction_inner,
actual = Datensatz_4_1$Hämoglobin)
```

```
MSE_DF_1.1

MSE_1.1<-mean((MSE_DF_1.1$actual - MSE_DF_1.1$pred)^2)

MSE_1.1

#ohne RE : 0.804068

#mit RE  : 0.8227654



### out-off sample performance 1###


Covariates.1.out <-
data.frame(1,Gruppe1[,names(Schätzer1[1:(length(Schätzer1)-
2)])[2:(length(Schätzer1)-2)]])

Covariates.1.out

Gruppe1$prediction_inner<-as.matrix(Covariates.1.out) %*%
as.matrix(Coefficients.1)

Gruppe1$prediction_inner

Gruppe1$prediction_inner <- ifelse(Gruppe1$prediction_inner < 0,
0, Gruppe1$prediction_inner)

Gruppe1$prediction_inner


### MSE berechnen 1###

MSE_DF_1.2 <- data.frame(pred = Gruppe1$prediction_inner, actual
= Gruppe1$Hämoglobin)

MSE_DF_1.2

MSE_1.2<-mean((MSE_DF_1.2$actual - MSE_DF_1.2$pred)^2)

MSE_1.2

#ohne RE : 3.248445

#mit RE  : 3.006627


### Differenz der MSEs 1###

Diff_tobit_1<-MSE_1.2-MSE_1.1

Diff_tobit_1

#ohne RE : 2.444377
```

```
#mit RE  : 2.183861

### Datensatz 2 ###

### fractional polynomials 1 ###

#df=4 !

model_fractional_2<- mfp(Hämoglobin ~fp(DT_EX1_342.41nm, df = 4,
select = 0.05) + fp( AT_EX2_363.92nm, df = 4, select = 0.05) +
fp( AR_EX2_363.92nm, df = 4,
                      select = 0.05)+  fp( AR_EX2_557.5nm, df =
4, select = 0.05) +  fp( DT_EX1_586.83nm, df = 4, select = 0.05),
data=Datensatz_4_2)


model_fractional_2



#Fractional polynomials:

#                 df.initial select alpha df.final power1 power2

#DT_EX1_586.83nm           4   0.05  0.05        4     -2     -2

#AT_EX2_363.92nm           4   0.05  0.05        0      .      .

#DT_EX1_342.41nm           4   0.05  0.05        0      .      .

#AR_EX2_557.5nm            4   0.05  0.05        1      1      .

#AR_EX2_363.92nm           4   0.05  0.05        0      .      .



#Transformations of covariates:

#
formula

#DT_EX1_342.41nm
<NA>

#AT_EX2_363.92nm
<NA>

#AR_EX2_363.92nm
<NA>

#AR_EX2_557.5nm
I(AR_EX2_557.5nm^1)
```

```
#DT_EX1_586.83nm I(DT_EX1_586.83nm^-2)+I(DT_EX1_586.83nm^-
2*log(DT_EX1_586.83nm))


#Datentransformation 2

#Trainingsdaten

Datensatz_4_2$AR_EX2_557.5nm_new<-
I(Datensatz_4_2$AR_EX2_557.5nm^1)

Datensatz_4_2$DT_EX1_586.83nm_new<-
I(Datensatz_4_2$DT_EX1_586.83nm^-
2)+I(Datensatz_4_2$DT_EX1_586.83nm^-
2*log(Datensatz_4_2$DT_EX1_586.83nm))


#Testdaten

Gruppe2$AR_EX2_557.5nm_new<-I(Gruppe2$AR_EX2_557.5nm^1)

Gruppe2$DT_EX1_586.83nm_new<-I(Gruppe2$DT_EX1_586.83nm^-
2)+I(Gruppe2$DT_EX1_586.83nm^-2*log(Gruppe2$DT_EX1_586.83nm))


#Tobit Modell mit RE für Datensatz_4_2

tobit_2<-censReg(Hämoglobin ~ AR_EX2_557.5nm_new +
DT_EX1_586.83nm_new, left = 0,

                right = Inf, data = Datensatz_4_2 ,
method="BHHH")

summary(tobit_2)

step(tobit_2,direction="backward") # entfernt

tobit_2<-censReg(Hämoglobin ~ DT_EX1_496.88nm_new +
AT_EX2_582.92nm_new, left = 0,

                 right = Inf, data = Datensatz_4_2,
method="BHHH")

summary(tobit_2)


#Geschätzte Koeffizienten als Schätzer definieren

Schätzer2<-tobit_2$estimate

Schätzer2

Covariates.2 <-
data.frame(1,Datensatz_4_2[,names(Schätzer2[1:(length(Schätzer2)
-2)])[2:(length(Schätzer2)-2)]])
```

```
Covariates.2

Coefficients.2 <- Schätzer2[1:(length(Schätzer2)-2)]

Coefficients.2

head(as.matrix(Covariates.2) %*% as.matrix(Coefficients.2))


#predicted inner

Datensatz_4_2$prediction_inner <- as.matrix(Covariates.2) %*%
as.matrix(Coefficients.2)

Datensatz_4_2$prediction_inner

Datensatz_4_2$prediction_inner <-
ifelse(Datensatz_4_2$prediction_inner < 0, 0,
Datensatz_4_2$prediction_inner)

Datensatz_4_2$prediction_inner


### MSE berechnen 2###

MSE_DF_2.1 <- data.frame(pred = Datensatz_4_2$prediction_inner,
actual = Datensatz_4_2$Hämoglobin)

MSE_2.1<-mean((MSE_DF_2.1$actual - MSE_DF_2.1$pred)^2)

MSE_2.1

#ohne RE : 1.289186

#mit RE  : 1.286119

### out-off sample performance 2###

Covariates.2.out <-
data.frame(1,Gruppe2[,names(Schätzer2[1:(length(Schätzer2)-
2)])[2:(length(Schätzer2)-2)]])

Covariates.2.out

Gruppe2$prediction_inner<-as.matrix(Covariates.2.out) %*%
as.matrix(Coefficients.2)

Gruppe2$prediction_inner

Gruppe2$prediction_inner <- ifelse(Gruppe2$prediction_inner < 0,
0, Gruppe2$prediction_inner)

Gruppe2$prediction_inner


### MSE berechnen 2###
```

```
MSE_DF_2.2 <- data.frame(pred = Gruppe2$prediction_inner, actual = Gruppe2$Hämoglobin)

MSE_2.2<-mean((MSE_DF_2.2$actual - MSE_DF_2.2$pred)^2)

MSE_2.2

#ohne RE : 0.9723

#mit RE :  0.5880088

### Differenz der MSEs 2###

Diff_tobit_2<-MSE_2.2-MSE_2.1

Diff_tobit_2

#ohne RE : -0.3168857

#mit RE :  -0.6981098

### Datensatz 3 ###

### fractional polynomials 3 ###

#df=2 !

model_fractional_3<- mfp(Hämoglobin ~fp(DT_EX1_342.41nm, df = 4, select = 0.05) + fp( AT_EX2_363.92nm, df = 4, select = 0.05) + fp( AR_EX2_363.92nm, df = 4,

                        select = 0.05)+  fp( AR_EX2_557.5nm, df = 4, select = 0.05) +  fp( DT_EX1_586.83nm, df = 4, select = 0.05), data=Datensatz_4_3)


model_fractional_3




#Fractional polynomials:

#                 df.initial select alpha df.final power1 power2

#DT_EX1_586.83nm          4   0.05  0.05        4     -2     -2

#DT_EX1_342.41nm          4   0.05  0.05        0      .      .

#AT_EX2_363.92nm          4   0.05  0.05        0      .      .

#AR_EX2_557.5nm           4   0.05  0.05        2     -2      .

#AR_EX2_363.92nm          4   0.05  0.05        0      .      .
```

```
#Transformations of covariates:

#
formula

#DT_EX1_342.41nm
<NA>

#AT_EX2_363.92nm
<NA>

#AR_EX2_363.92nm
<NA>

#AR_EX2_557.5nm
I(AR_EX2_557.5nm^-2)

#DT_EX1_586.83nm I(DT_EX1_586.83nm^-2)+I(DT_EX1_586.83nm^-
2*log(DT_EX1_586.83nm))




#Datentransformation 3

Datensatz_4_3$AR_EX2_557.5nm_new<-
I(Datensatz_4_3$AR_EX2_557.5nm^-2)

Datensatz_4_3$DT_EX1_586.83nm_new<-
I(Datensatz_4_3$DT_EX1_586.83nm^-
2)+I(Datensatz_4_3$DT_EX1_586.83nm^-
2*log(Datensatz_4_3$DT_EX1_586.83nm))


Gruppe3$AR_EX2_557.5nm_new<- I(Gruppe3$AR_EX2_557.5nm^-2)

Gruppe3$DT_EX1_586.83nm_new<- I(Gruppe3$DT_EX1_586.83nm^-
2)+I(Gruppe3$DT_EX1_586.83nm^-2*log(Gruppe3$DT_EX1_586.83nm))

tobit_3<-censReg(Hämoglobin ~ AR_EX2_557.5nm_new +
DT_EX1_586.83nm_new, left = 0,

                 right = Inf, data = Datensatz_4_3 ,
method="BHHH")

summary(tobit_3)

step(tobit_3,direction="backward")

tobit_3<-censReg(Hämoglobin ~ AR_EX2_557.5nm_new +
DT_EX1_586.83nm_new, left = 0,

                 right = Inf, data = Datensatz_4_3 ,
method="BHHH")
```

```
summary(tobit_3)


#Geschätzte Koeffizienten als Schätzer definieren

Schätzer3<-tobit_3$estimate

Schätzer3

Covariates.3 <-
data.frame(1,Datensatz_4_3[,names(Schätzer3[1:(length(Schätzer3)
-2)])[2:(length(Schätzer3)-2)]])

Covariates.3

Coefficients.3 <- Schätzer3[1:(length(Schätzer3)-2)]

Coefficients.3

head(as.matrix(Covariates.3) %*% as.matrix(Coefficients.3))


#predicted inner

Datensatz_4_3$prediction_inner <- as.matrix(Covariates.3) %*%
as.matrix(Coefficients.3)

Datensatz_4_3$prediction_inner


Datensatz_4_3$prediction_inner <-
ifelse(Datensatz_4_3$prediction_inner < 0, 0,
Datensatz_4_3$prediction_inner)

Datensatz_4_3$prediction_inner

### MSE berechnen 3###

MSE_DF_3.1 <- data.frame(pred = Datensatz_4_3$prediction_inner,
actual = Datensatz_4_3$Hämoglobin)

MSE_DF_3.1

MSE_3.1<-mean((MSE_DF_3.1$actual - MSE_DF_3.1$pred)^2)

MSE_3.1

#ohne RE : 1.041074

#mit RE  : 0.9917713

### out-off sample performance 3###

Covariates.3.out <-
data.frame(1,Gruppe3[,names(Schätzer3[1:(length(Schätzer3)-
2)])[2:(length(Schätzer3)-2)]])
```

```
Covariates.3.out

Gruppe3$prediction_inner<-as.matrix(Covariates.3.out) %*%
as.matrix(Coefficients.3)

Gruppe3$prediction_inner

Gruppe3$prediction_inner <- ifelse(Gruppe3$prediction_inner < 0,
0, Gruppe3$prediction_inner)

Gruppe3$prediction_inner

### MSE berechnen 3###

MSE_DF_3.2 <- data.frame(pred = Gruppe3$prediction_inner, actual
= Gruppe3$Hämoglobin)

MSE_3.2<-mean((MSE_DF_3.2$actual - MSE_DF_3.2$pred)^2)

MSE_3.2

#ohne RE : 2.330614

#mit RE :  1.682918

### Differenz der MSEs 3###

Diff_tobit_3<-MSE_3.2-MSE_3.1

Diff_tobit_3

#ohne RE :  1.28954

#mit RE :  0.6911464

### Datensatz 4 ###

### fractional polynomials 4 ###

#df=2 !

model_fractional_4<- mfp(Hämoglobin ~fp(DT_EX1_342.41nm, df = 4,
select = 0.05) + fp( AT_EX2_363.92nm, df = 4, select = 0.05) +
fp( AR_EX2_363.92nm, df = 4,

                          select = 0.05)+  fp( AR_EX2_557.5nm,
df = 4, select = 0.05) +  fp( DT_EX1_586.83nm, df = 4, select =
0.05) , data=Datensatz_4_4)


model_fractional_4




#Fractional polynomials:

#                df.initial select alpha df.final power1 power2
```

```
#DT_EX1_586.83nm          4   0.05  0.05        4     -2     -2

#AT_EX2_363.92nm          4   0.05  0.05        0      .      .

#DT_EX1_342.41nm          4   0.05  0.05        0      .      .

#AR_EX2_557.5nm           4   0.05  0.05        1      1      .

#AR_EX2_363.92nm          4   0.05  0.05        0      .      .



#Transformations of covariates:

#
formula

#DT_EX1_342.41nm
<NA>

#AT_EX2_363.92nm
<NA>

#AR_EX2_363.92nm
<NA>

#AR_EX2_557.5nm
I(AR_EX2_557.5nm^1)

#DT_EX1_586.83nm I(DT_EX1_586.83nm^-2)+I(DT_EX1_586.83nm^-
2*log(DT_EX1_586.83nm))


#Datentransformation 4

#Trainingsdaten

Datensatz_4_4$AR_EX2_557.5nm_new<-
I(Datensatz_4_4$AR_EX2_557.5nm^1)

Datensatz_4_4$DT_EX1_586.83nm_new<-
I(Datensatz_4_4$DT_EX1_586.83nm^-
2)+I(Datensatz_4_4$DT_EX1_586.83nm^-
2*log(Datensatz_4_4$DT_EX1_586.83nm))

#Testdaten

Gruppe4$AR_EX2_557.5nm_new<- I(Gruppe4$AR_EX2_557.5nm^1)

Gruppe4$DT_EX1_586.83nm_new<- I(Gruppe4$DT_EX1_586.83nm^-
2)+I(Gruppe4$DT_EX1_586.83nm^-2*log(Gruppe4$DT_EX1_586.83nm))


tobit_4<-censReg(Hämoglobin ~ AR_EX2_557.5nm_new +
DT_EX1_586.83nm_new, left = 0,
```

```
                 right = Inf, data = Datensatz_4_4 ,
method="BHHH")


summary(tobit_4)

step(tobit_4,direction="backward") # entfernt

tobit_4<-censReg(Hämoglobin ~ AR_EX2_557.5nm_new +
DT_EX1_586.83nm_new, left = 0,

                 right = Inf, data = Datensatz_4_4 ,
method="BHHH")

summary(tobit_4)

#Geschätzte Koeffizienten als Schätzer definieren

Schätzer4<-tobit_4$estimate

Schätzer4

Covariates.4 <-
data.frame(1,Datensatz_4_4[,names(Schätzer4[1:(length(Schätzer4)
-2)])[2:(length(Schätzer4)-2)]])

Covariates.4

Coefficients.4 <- Schätzer4[1:(length(Schätzer4)-2)]

Coefficients.4

head(as.matrix(Covariates.4) %*% as.matrix(Coefficients.4))


#predicted inner

Datensatz_4_4$prediction_inner <- as.matrix(Covariates.4) %*%
as.matrix(Coefficients.4)

Datensatz_4_4$prediction_inner


Datensatz_4_4$prediction_inner <-
ifelse(Datensatz_4_4$prediction_inner < 0, 0,
Datensatz_4_4$prediction_inner)

Datensatz_4_4$prediction_inner


### MSE berechnen 4###

MSE_DF_4.1 <- data.frame(pred = Datensatz_4_4$prediction_inner,
actual = Datensatz_4_4$Hämoglobin)

MSE_4.1<-mean((MSE_DF_4.1$actual - MSE_DF_4.1$pred)^2)
```

```
MSE_4.1

#ohne RE : 1.304894

#mit RE :  1.281395


### out-off sample performance 4###

Covariates.4.out <-
data.frame(1,Gruppe4[,names(Schätzer4[1:(length(Schätzer4)-
2)])[2:(length(Schätzer4)-2)]])

Covariates.4.out

Gruppe4$prediction_inner<-as.matrix(Covariates.4.out) %*%
as.matrix(Coefficients.4)

Gruppe4$prediction_inner

Gruppe4$prediction_inner <- ifelse(Gruppe4$prediction_inner < 0,
0, Gruppe4$prediction_inner)

Gruppe4$prediction_inner


### MSE berechnen 4###

MSE_DF_4.2 <- data.frame(pred = Gruppe4$prediction_inner, actual
= Gruppe4$Hämoglobin)

MSE_4.2<-mean((MSE_DF_4.2$actual - MSE_DF_4.2$pred)^2)

MSE_4.2

#ohne RE : 0.9843887

#mit RE :  0.624709

### Differenz der MSEs 4###

Diff_tobit_4<-MSE_4.2-MSE_4.1

Diff_tobit_4

#ohne RE : -0.320505

#mit RE :  -0.6566857


### Datensatz 5 ###

### fractional polynomials 5 ###

#df=2 !
```

```
model_fractional_5<- mfp(Hämoglobin ~fp(DT_EX1_342.41nm, df = 4,
select = 0.05) + fp( AT_EX2_363.92nm, df = 4, select = 0.05) +
fp( AR_EX2_363.92nm, df = 4,

                              select = 0.05)+
fp( AR_EX2_557.5nm, df = 4, select = 0.05) +
fp( DT_EX1_586.83nm, df = 4, select = 0.05) , data=Datensatz_4_5)

model_fractional_5

#Fractional polynomials:

#                df.initial select alpha df.final power1 power2

#AT_EX2_582.92nm          4   0.05  0.05        4     -2     -2

#DT_EX1_496.88nm          4   0.05  0.05        4     -2     -1

#DT_EX1_745.22nm          4   0.05  0.05        4      3      3

#Transformations of covariates:

#
formula

#DT_EX1_496.88nm                     I(DT_EX1_496.88nm^-
2)+I(DT_EX1_496.88nm^-1)

#AT_EX2_582.92nm I(AT_EX2_582.92nm^-2)+I(AT_EX2_582.92nm^-
2*log(AT_EX2_582.92nm))

#DT_EX1_745.22nm
I(DT_EX1_745.22nm^3)+I(DT_EX1_745.22nm^3*log(DT_EX1_745.22nm))

#Datentransformation 5

#Trainingsdaten

Datensatz_4_5$AR_EX2_557.5nm_new <-
I(Datensatz_4_5$AR_EX2_557.5nm^1)

Datensatz_4_5$DT_EX1_586.83nm_new <-
I(Datensatz_4_5$DT_EX1_586.83nm^-
2)+I(Datensatz_4_5$DT_EX1_586.83nm^-
2*log(Datensatz_4_5$DT_EX1_586.83nm))

#Testdaten

Gruppe5$AR_EX2_557.5nm_new <-I(Gruppe5$AR_EX2_557.5nm^1)

Gruppe5$DT_EX1_586.83nm_new <-I(Gruppe5$DT_EX1_586.83nm^-
2)+I(Gruppe5$DT_EX1_586.83nm^-2*log(Gruppe5$DT_EX1_586.83nm)

tobit_5<-censReg(Hämoglobin ~ AR_EX2_557.5nm_new +
DT_EX1_586.83nm_new, left = 0,

                 right = Inf, data = Datensatz_4_5 ,
method="BHHH")
```

```
tobit_5<- survreg(Surv(Hämoglobin, Hämoglobin>0, type='left') ~ AR_EX2_557.5nm_new + DT_EX1_586.83nm_new

                      ,data=Datensatz_4_5, dist='gaussian')

summary(tobit_5)

step(tobit_5,direction="backward") #AR_EX2_557.5nm_new wird entfernt


tobit_5<-censReg(Hämoglobin ~ DT_EX1_586.83nm_new , left = 0,

                 right = Inf, data = Datensatz_4_5 , method="BHHH")

summary(tobit_5)


#Geschätzte Koeffizienten als Schätzer definieren

Schätzer5<-tobit_5$estimate

Schätzer5

Covariates.5 <- data.frame(1,Datensatz_4_5[,names(Schätzer5[1:(length(Schätzer5)-2)])[2:(length(Schätzer5)-2)]])

Covariates.5

Coefficients.5 <- Schätzer5[1:(length(Schätzer5)-2)]

Coefficients.5

head(as.matrix(Covariates.5) %*% as.matrix(Coefficients.5))


#predicted innerDatensatz_4_5$prediction_inner <- as.matrix(Covariates.5) %*% as.matrix(Coefficients.5)

#Datensatz_4_5$prediction_inner

Datensatz_4_5$prediction_inner <- ifelse(Datensatz_4_5$prediction_inner < 0, 0, Datensatz_4_5$prediction_inner)

Datensatz_4_5$prediction_inner



### MSE berechnen 5###
```

```
MSE_DF_5.1 <- data.frame(pred = Datensatz_4_5$prediction_inner,
actual = Datensatz_4_5$Hämoglobin)

MSE_5.1<-mean((MSE_DF_5.1$actual - MSE_DF_5.1$pred)^2)

MSE_5.1

#ohne RE : 1.332092

#mit RE  : 1.365008

### out-off sample performance 4###

Covariates.5.out <-
data.frame(1,Gruppe5[,names(Schätzer5[1:(length(Schätzer5)-
2)])[2:(length(Schätzer5)-2)]])

Covariates.5.out

Gruppe5$prediction_inner<-as.matrix(Covariates.5.out) %*%
as.matrix(Coefficients.5)

Gruppe5$prediction_inner

Gruppe5$prediction_inner <- ifelse(Gruppe5$prediction_inner < 0,
0, Gruppe5$prediction_inner)

Gruppe5$prediction_inner

### MSE berechnen 5###

MSE_DF_5.2 <- data.frame(pred = Gruppe5$prediction_inner, actual
= Gruppe5$Hämoglobin)

MSE_5.2<-mean((MSE_DF_5.2$actual - MSE_DF_5.2$pred)^2)

MSE_5.2

#ohne RE : 0.9534782

#mit RE  : 0.2642826

### Differenz der MSEs 5###

Diff_tobit_5<-MSE_5.2-MSE_5.1

Diff_tobit_5

#ohne RE : -0.3786136

#mit RE  : -1.100726




### Datensatz gesamt ###
```

```
### fractional polynomials gesamt ###

#df=2 !

model_fractional_gesamt<- mfp(Hämoglobin ~fp(DT_EX1_342.41nm, df
= 4, select = 0.05) + fp( AT_EX2_363.92nm, df = 4, select = 0.05)
+  fp( AR_EX2_363.92nm, df = 4,

                              select = 0.05)+
fp( AR_EX2_557.5nm, df = 4, select = 0.05) +
fp( DT_EX1_586.83nm, df = 4, select = 0.05) , data=Hämoglobin)

model_fractional_gesamt


#Fractional polynomials:

#               df.initial select alpha df.final power1 power2

#DT_EX1_586.83nm          4   0.05  0.05        4     -2     -2

#DT_EX1_342.41nm          4   0.05  0.05        0      .      .

#AT_EX2_363.92nm          4   0.05  0.05        1      1      .

#AR_EX2_557.5nm           4   0.05  0.05        1      1      .

#AR_EX2_363.92nm          4   0.05  0.05        0      .      .


#Transformations of covariates:

#
formula

#DT_EX1_342.41nm
<NA>

#AT_EX2_363.92nm
I((AT_EX2_363.92nm/10)^1)

#AR_EX2_363.92nm
<NA>

#  AR_EX2_557.5nm
I(AR_EX2_557.5nm^1)

#DT_EX1_586.83nm I(DT_EX1_586.83nm^-2)+I(DT_EX1_586.83nm^-
2*log(DT_EX1_586.83nm))


Hämoglobin$AT_EX2_363.92nm_new<-
I((Hämoglobin$AT_EX2_363.92nm/10)^1)

Hämoglobin$AR_EX2_557.5nm_new<- I(Hämoglobin$AR_EX2_557.5nm^1)
```

```
Hämoglobin$DT_EX1_586.83nm_new<- I(Hämoglobin$DT_EX1_586.83nm^-
2)+I(Hämoglobin$DT_EX1_586.83nm^-
2*log(Hämoglobin$DT_EX1_586.83nm))




tobit_gesamt<-censReg(Hämoglobin ~ AT_EX2_363.92nm_new +
AR_EX2_557.5nm_new +DT_EX1_586.83nm_new, left = 0,
                     right = Inf, data = Hämoglobin,
method="BHHH")

summary(tobit_gesamt)

step(tobit_gesamt,direction="backward") #AR_EX2_557.5nm_new wird
entfernt

tobit_gesamt<-censReg(Hämoglobin ~ AT_EX2_363.92nm_new +
DT_EX1_586.83nm_new , left = 0,
                      right = Inf, data = Hämoglobin ,
method="BHHH")

summary(tobit_gesamt)

#Geschätzte Koeffizienten als Schätzer definieren

Schätzer_gesamt<-tobit_gesamt$estimate

Schätzer_gesamt

#Covariates.gesamt <-
data.frame(1,Hämoglobin[,names(Schätzer_gesamt[1:(length(Schätze
r_gesamt)-2)])[2:(length(Schätzer_gesamt)-2)]])

Covariates.gesamt

Coefficients.gesamt <-
Schätzer_gesamt[1:(length(Schätzer_gesamt)-2)]

Coefficients.gesamt

head(as.matrix(Covariates.gesamt) %*%
as.matrix(Coefficients.gesamt))


#predicted inner

Hämoglobin$prediction_inner <- as.matrix(Covariates.gesamt) %*%
as.matrix(Coefficients.gesamt)

Hämoglobin$prediction_inner
```

```
Hämoglobin$prediction_inner <-
ifelse(Hämoglobin$prediction_inner < 0, 0,
Hämoglobin$prediction_inner)

Hämoglobin$prediction_inner

### MSE berechnen gesamr###

MSE_DF_gesamt <- data.frame(pred = Hämoglobin$prediction_inner,
actual = Hämoglobin$Hämoglobin)


MSE_gesamt<-mean((MSE_DF_gesamt$actual - MSE_DF_gesamt$pred)^2)

MSE_gesamt

#ohne RE : 1.103596

#mit RE :  1.1032


library(ggplot2)

ggplot(MSE_DF_gesamt, aes(x = actual, y = pred)) +

  geom_point(alpha = 0.5, color = 'blue') +

  labs(title = 'Actual vs. Predicted Values',

       x = 'Actual Values',

       y = 'Predicted Values') +

  # Calculate and plot the error as vertical lines

  geom_segment(aes(x = actual, xend = actual, y = actual, yend =
pred), color = 'red', linetype = 'dashed') +

  # Set log scale for both x and y axes

  #scale_x_log10() +

  #scale_y_log10() +

  xlim(c(min(MSE_DF_gesamt$actual), max(MSE_DF_gesamt$actual)))
+

  ylim(c(min(MSE_DF_gesamt$actual), max(MSE_DF_gesamt$actual)))
+

  theme_minimal()


### Mittelwert der 5 MSE-Differenzen berechnen ###

Unterschied<-(Diff_tobit_1 + Diff_tobit_2 + Diff_tobit_3 +
Diff_tobit_4 + Diff_tobit_5)/5
```

```
Unterschied
#ohne RE : 0.5435825
#mit RE :  0.08389727


MSE_global_corrected<-MSE_gesamt + Unterschied
MSE_global_corrected
#ohne RE : 1.647179
#mit RE :  1.187097
#mit RE ohne predicted values=0 :

var(Hämoglobin$Hämoglobin)
#3.291052
```
