# Supplementary material for: Intelligent surgical drainage - digitizing the analysis of drainage fluid in patients with surgical drains
Source: PLoS One. 2025 Jul 28;20(7):e0325072. doi: 10.1371/journal.pone.0325072 (PMC12303269; doi:10.1371/journal.pone.0325072)
Supplement: S2 File — (PDF) [file pone.0325072.s010.pdf]

```
#install.packages("ISLR")
library(ISLR)
#install.packages("caret")
library(caret)
#install.packages("glmtoolbox")
library(glmtoolbox)
#install.packages("dplyr")
library(dplyr)
#install.packages("ConfusionTableR")
library(ConfusionTableR)
#install.packages("tidyr")
library(tidyr)
#install.packages("mlbench")
library(mlbench)
#install.packages("mfp") #wird fÃ¼r fractional polynomial 
benÃ¶tigt
library(mfp)
#working directory
setwd("F:/1. Klinisches Semester/Promotion/R Analyse")
#Daten werden eingelesen
#install.packages("readxl")
library(readxl)
#install.packages("olsrr")     #wird fÃ¼r die model selection 
benÃ¶tigt
library(olsrr)
#install.packages("survival")
library(survival)
#install.packages("censReg")
library(censReg)
#install.packages("plm") #wird für die Berücksichtigung von 
Random Effects benötigt
library(plm)
```

```
########################################################################
######

Hämoglobin <- read_excel("7_hemoglobin input dataset for R.xlsx")

hist(Hämoglobin$DT_EX1_342.41nm)

hist(Hämoglobin$AT_EX2_363.92nm)

hist(Hämoglobin$AR_EX2_363.92nm)

hist(Hämoglobin$AR_EX2_557.5nm)

hist(Hämoglobin$DT_EX1_586.83nm)


min (Hämoglobin$DT_EX1_342.41nm)

#-0.9256574

min(Hämoglobin$AT_EX2_363.92nm)

#-1.054466

min(Hämoglobin$AR_EX2_363.92nm)

#-1.011524

min(Hämoglobin$AR_EX2_557.5nm)

#-0.9856557

min(Hämoglobin$DT_EX1_586.83nm)

#-0.7216694


########################################################################
########################################################################
###################################

### Einführung einer künstlichen unteren Grenze der Regressoren
###

Hämoglobin$DT_EX1_342.41nm <- ifelse(Hämoglobin$DT_EX1_342.41nm
< -2, -2, Hämoglobin$DT_EX1_342.41nm)

Hämoglobin$AT_EX2_363.92nm <- ifelse(Hämoglobin$AT_EX2_363.92nm
< -2, -2, Hämoglobin$AT_EX2_363.92nm)

Hämoglobin$AR_EX2_363.92nm <- ifelse(Hämoglobin$AR_EX2_363.92nm
< -2, -2, Hämoglobin$AR_EX2_363.92nm)

Hämoglobin$AR_EX2_557.5nm <- ifelse(Hämoglobin$AR_EX2_557.5nm <
-2, -2, Hämoglobin$AR_EX2_557.5nm)
```

```
Hämoglobin$DT_EX1_586.83nm <- ifelse(Hämoglobin$DT_EX1_586.83nm
< -2, -2, Hämoglobin$DT_EX1_586.83nm)


#Addieren einer Konstanten, damit keine negativen Regressorwerte
im Testdatensatz auftauchen

Hämoglobin$DT_EX1_342.41nm<-Hämoglobin$DT_EX1_342.41nm+2.1

Hämoglobin$AT_EX2_363.92nm<-Hämoglobin$AT_EX2_363.92nm+2.1

Hämoglobin$AR_EX2_363.92nm<-Hämoglobin$AR_EX2_363.92nm+2.1

Hämoglobin$AR_EX2_557.5nm<-Hämoglobin$AR_EX2_557.5nm+2.1

Hämoglobin$DT_EX1_586.83nm<-Hämoglobin$DT_EX1_586.83nm+2.1


#################################################

### Aufteilen des Datensatzes in 5 Teilmengen ###

#################################################

set.seed(2023)

sample_size <- nrow(Hämoglobin)

set_proportions <- c(Gruppe1 = 0.2, Gruppe2 = 0.2, Gruppe3 = 0.2,
Gruppe4= 0.2, Gruppe5=0.2)

set_frequencies <- diff(floor(sample_size * cumsum(c(0,
set_proportions))))

Hämoglobin$set <- sample(rep(names(set_proportions), times =
set_frequencies))

#Erstellen der 5 subsamples

Gruppe1 <- Hämoglobin[Hämoglobin$set == "Gruppe1", ]

Gruppe2 <- Hämoglobin[Hämoglobin$set == "Gruppe2", ]

Gruppe3 <- Hämoglobin[Hämoglobin$set == "Gruppe3", ]

Gruppe4 <- Hämoglobin[Hämoglobin$set == "Gruppe4", ]

Gruppe5 <- Hämoglobin[Hämoglobin$set == "Gruppe5", ]


#Definiere die 5 DatensÃ¤tze, an denen die Schritte 3
durchgeführt werden

Datensatz_4_1 <- rbind(Gruppe2, Gruppe3, Gruppe4, Gruppe5)

Datensatz_4_2 <- rbind(Gruppe1, Gruppe3, Gruppe4, Gruppe5)
```

```
Datensatz_4_3 <- rbind(Gruppe1, Gruppe2, Gruppe4, Gruppe5)

Datensatz_4_4 <- rbind(Gruppe1, Gruppe2, Gruppe3, Gruppe5)

Datensatz_4_5 <- rbind(Gruppe1, Gruppe2, Gruppe3, Gruppe4)

### Datensatz 1 ###

###################


### fractional polynomials 1 ###

#df=4 !

model_fractional_1<- mfp(Hämoglobin ~fp( DT_EX1_342.41nm, df = 4,
select = 0.05) + fp( AT_EX2_363.92nm, df = 4, select = 0.05) +
fp( AR_EX2_363.92nm, df = 4,

                                  select = 0.05)+
fp( AR_EX2_557.5nm, df = 4, select = 0.05) +
fp( DT_EX1_586.83nm, df = 4, select = 0.05) , data=Datensatz_4_1)

model_fractional_1


#Datentransformation 1

#Trainingsdaten

Datensatz_4_1$AT_EX2_363.92nm_new<-
I((Datensatz_4_1$AT_EX2_363.92nm/10)^1)

Datensatz_4_1$AR_EX2_557.5nm_new<-
I(Datensatz_4_1$AR_EX2_557.5nm^1)

Datensatz_4_1$DT_EX1_586.83nm_new<-
I(Datensatz_4_1$DT_EX1_586.83nm^-
2)+I(Datensatz_4_1$DT_EX1_586.83nm^-
2*log(Datensatz_4_1$DT_EX1_586.83nm))


#Testdaten

Gruppe1$AT_EX2_363.92nm_new<-I((Gruppe1$AT_EX2_363.92nm/10)^1)

Gruppe1$AR_EX2_557.5nm_new<-I(Gruppe1$AR_EX2_557.5nm^1)

Gruppe1$DT_EX1_586.83nm_new<-I(Gruppe1$DT_EX1_586.83nm^-
2)+I(Gruppe1$DT_EX1_586.83nm^-2*log(Gruppe1$DT_EX1_586.83nm))

#tobit ohne RE

tobit_1 <- survreg(Surv(Hämoglobin, Hämoglobin>0, type='left') ~
AT_EX2_363.92nm_new + AR_EX2_557.5nm_new + DT_EX1_586.83nm_new
```

```
                   ,data=Datensatz_4_1, dist='gaussian')


#backward selection

step(tobit_1,direction="backward") #AR_EX2_557.5nm_new  wird
entfernt


summary(tobit_1)

tobit_1 <- survreg(Surv(Hämoglobin, Hämoglobin>0, type='left') ~
AT_EX2_363.92nm_new + DT_EX1_586.83nm_new

                   ,data=Datensatz_4_1, dist='gaussian')

summary(tobit_1)

# predicted values ohne RE 1

Datensatz_4_1$prediction_inner <-
predict(tobit_1,type="response")

Datensatz_4_1$prediction_inner


Datensatz_4_1$prediction_inner <-
ifelse(Datensatz_4_1$prediction_inner < 0, 0,
Datensatz_4_1$prediction_inner)

Datensatz_4_1$prediction_inner

### MSE berechnen 1###

MSE_DF_1.1 <- data.frame(pred=Datensatz_4_1$prediction_inner,
actual = Datensatz_4_1$Hämoglobin)

MSE_DF_1.1

MSE_1.1<-mean((MSE_DF_1.1$actual - MSE_DF_1.1$pred)^2)

MSE_1.1

#ohne RE : 0.804068

# out-off sample performance 1 ohne RE###

Gruppe1$prediction_inner <- predict(tobit_1,newdata = Gruppe1)

Gruppe1$prediction_inner

### MSE berechnen 1###

MSE_DF_1.2 <- data.frame(pred = Gruppe1$prediction_inner, actual
= Gruppe1$Hämoglobin)

MSE_DF_1.2
```

```
MSE_1.2<-mean((MSE_DF_1.2$actual - MSE_DF_1.2$pred)^2)

MSE_1.2

#ohne RE : 3.248445

#mit RE  : 3.006627

### Differenz der MSEs 1###

Diff_tobit_1<-MSE_1.2-MSE_1.1

Diff_tobit_1

#ohne RE : 2.444377

#mit RE  : 2.183861


### Datensatz 2 ###

### fractional polynomials 1 ###

#df=4 !

model_fractional_2<- mfp(Hämoglobin ~fp(DT_EX1_342.41nm, df = 4, 
select = 0.05) + fp( AT_EX2_363.92nm, df = 4, select = 0.05) + 
fp( AR_EX2_363.92nm, df = 4,

                      select = 0.05)+  fp( AR_EX2_557.5nm, df = 
4, select = 0.05) +  fp( DT_EX1_586.83nm, df = 4, select = 0.05), 
data=Datensatz_4_2)

model_fractional_2

#Datentransformation 2

#Trainingsdaten

Datensatz_4_2$AR_EX2_557.5nm_new<-
I(Datensatz_4_2$AR_EX2_557.5nm^1)

Datensatz_4_2$DT_EX1_586.83nm_new<-
I(Datensatz_4_2$DT_EX1_586.83nm^-
2)+I(Datensatz_4_2$DT_EX1_586.83nm^-
2*log(Datensatz_4_2$DT_EX1_586.83nm))

#Testdaten

Gruppe2$AR_EX2_557.5nm_new<-I(Gruppe2$AR_EX2_557.5nm^1)

Gruppe2$DT_EX1_586.83nm_new<-I(Gruppe2$DT_EX1_586.83nm^-
2)+I(Gruppe2$DT_EX1_586.83nm^-2*log(Gruppe2$DT_EX1_586.83nm))

tobit_2 <- survreg(Surv(Hämoglobin, Hämoglobin>0, type='left') ~ 
AR_EX2_557.5nm_new + DT_EX1_586.83nm_new

                     ,data=Datensatz_4_2, dist='gaussian')
```

```
summary(tobit_2)

step(tobit_2,direction="backward")

tobit_2 <- survreg(Surv(Hämoglobin, Hämoglobin>0, type='left') ~ AR_EX2_557.5nm_new + DT_EX1_586.83nm_new

                     ,data=Datensatz_4_2, dist='gaussian')

summary(tobit_2)

# predicted values ohne RE

Datensatz_4_2$prediction_inner <- predict(tobit_2,type="response")

Datensatz_4_2$prediction_inner


Datensatz_4_2$prediction_inner <- ifelse(Datensatz_4_2$prediction_inner < 0, 0, Datensatz_4_2$prediction_inner)

Datensatz_4_2$prediction_inner

### MSE berechnen 2###

MSE_DF_2.1 <- data.frame(pred = Datensatz_4_2$prediction_inner, actual = Datensatz_4_2$Hämoglobin)

MSE_2.1<-mean((MSE_DF_2.1$actual - MSE_DF_2.1$pred)^2)

MSE_2.1

#ohne RE : 1.289186

#mit RE  : 1.286119

# out-off sample performance 2 ohne RE###

Gruppe2$prediction_inner <- predict(tobit_2,newdata = Gruppe2)

Gruppe2$prediction_inner

### MSE berechnen 2###

MSE_DF_2.2 <- data.frame(pred = Gruppe2$prediction_inner, actual = Gruppe2$Hämoglobin)

MSE_2.2<-mean((MSE_DF_2.2$actual - MSE_DF_2.2$pred)^2)

MSE_2.2

#ohne RE : 0.9723

#mit RE :  0.5880088

### Differenz der MSEs 2###
```

```
Diff_tobit_2<-MSE_2.2-MSE_2.1

Diff_tobit_2

#ohne RE : -0.3168857

#mit RE :  -0.6981098




### Datensatz 3 ###

### fractional polynomials 3 ###

#df=2 !

model_fractional_3<- mfp(Hämoglobin ~fp(DT_EX1_342.41nm, df = 4, select = 0.05) + fp( AT_EX2_363.92nm, df = 4, select = 0.05) + fp( AR_EX2_363.92nm, df = 4,

                        select = 0.05)+  fp( AR_EX2_557.5nm, df = 4, select = 0.05) +  fp( DT_EX1_586.83nm, df = 4, select = 0.05), data=Datensatz_4_3)


model_fractional_3

#Datentransformation 3

Datensatz_4_3$AR_EX2_557.5nm_new<- I(Datensatz_4_3$AR_EX2_557.5nm^-2)

Datensatz_4_3$DT_EX1_586.83nm_new<- I(Datensatz_4_3$DT_EX1_586.83nm^-2)+I(Datensatz_4_3$DT_EX1_586.83nm^-2*log(Datensatz_4_3$DT_EX1_586.83nm))


Gruppe3$AR_EX2_557.5nm_new<- I(Gruppe3$AR_EX2_557.5nm^-2)

Gruppe3$DT_EX1_586.83nm_new<- I(Gruppe3$DT_EX1_586.83nm^-2)+I(Gruppe3$DT_EX1_586.83nm^-2*log(Gruppe3$DT_EX1_586.83nm))

tobit_3 <- survreg(Surv(Hämoglobin, Hämoglobin>0, type='left') ~ AR_EX2_557.5nm_new + DT_EX1_586.83nm_new

                     ,data=Datensatz_4_3, dist='gaussian')

summary(tobit_3)

step(tobit_3,direction="backward")

tobit_3 <- survreg(Surv(Hämoglobin, Hämoglobin>0, type='left') ~ AR_EX2_557.5nm_new + DT_EX1_586.83nm_new
```

```
                     ,data=Datensatz_4_3, dist='gaussian')

summary(tobit_3)

# predicted values ohne RE

Datensatz_4_3$prediction_inner <-
predict(tobit_3,type="response")

Datensatz_4_3$prediction_inner


Datensatz_4_3$prediction_inner <-
ifelse(Datensatz_4_3$prediction_inner < 0, 0,
Datensatz_4_3$prediction_inner)

Datensatz_4_3$prediction_inner

### MSE berechnen 3###

MSE_DF_3.1 <- data.frame(pred = Datensatz_4_3$prediction_inner,
actual = Datensatz_4_3$Hämoglobin)

MSE_DF_3.1

MSE_3.1<-mean((MSE_DF_3.1$actual - MSE_DF_3.1$pred)^2)

MSE_3.1

#ohne RE : 1.041074

#mit RE  : 0.9917713

# out-off sample performance 3 ohne RE###

Gruppe3$prediction_inner <- predict(tobit_3,newdata = Gruppe3)

Gruppe3$prediction_inner

### MSE berechnen 3###

MSE_DF_3.2 <- data.frame(pred = Gruppe3$prediction_inner, actual
= Gruppe3$Hämoglobin)

MSE_3.2<-mean((MSE_DF_3.2$actual - MSE_DF_3.2$pred)^2)

MSE_3.2

#ohne RE : 2.330614

#mit RE :  1.682918

### Differenz der MSEs 3###

Diff_tobit_3<-MSE_3.2-MSE_3.1

Diff_tobit_3

#ohne RE :  1.28954
```

```
#mit RE :  0.6911464


### Datensatz 4 ###

### fractional polynomials 4 ###

#df=2 !

model_fractional_4<- mfp(Hämoglobin ~fp(DT_EX1_342.41nm, df = 4,
select = 0.05) + fp( AT_EX2_363.92nm, df = 4, select = 0.05) +
fp( AR_EX2_363.92nm, df = 4,

                          select = 0.05)+  fp( AR_EX2_557.5nm,
df = 4, select = 0.05) +  fp( DT_EX1_586.83nm, df = 4, select =
0.05) , data=Datensatz_4_4)


model_fractional_4

#Datentransformation 4

#Trainingsdaten

Datensatz_4_4$AR_EX2_557.5nm_new<-
I(Datensatz_4_4$AR_EX2_557.5nm^1)

Datensatz_4_4$DT_EX1_586.83nm_new<-
I(Datensatz_4_4$DT_EX1_586.83nm^-
2)+I(Datensatz_4_4$DT_EX1_586.83nm^-
2*log(Datensatz_4_4$DT_EX1_586.83nm))

#Testdaten

Gruppe4$AR_EX2_557.5nm_new<- I(Gruppe4$AR_EX2_557.5nm^1)

Gruppe4$DT_EX1_586.83nm_new<- I(Gruppe4$DT_EX1_586.83nm^-
2)+I(Gruppe4$DT_EX1_586.83nm^-2*log(Gruppe4$DT_EX1_586.83nm))

tobit_4<- survreg(Surv(Hämoglobin, Hämoglobin>0, type='left') ~
AR_EX2_557.5nm_new + DT_EX1_586.83nm_new

                       ,data=Datensatz_4_4, dist='gaussian')

summary(tobit_4)

step(tobit_4,direction="backward")

tobit_4<- survreg(Surv(Hämoglobin, Hämoglobin>0, type='left') ~
AR_EX2_557.5nm_new + DT_EX1_586.83nm_new

                       ,data=Datensatz_4_4, dist='gaussian')

summary(tobit_4)
```

```
# predicted values ohne RE

Datensatz_4_4$prediction_inner <-
predict(tobit_4,type="response")

Datensatz_4_4$prediction_inner


Datensatz_4_4$prediction_inner <-
ifelse(Datensatz_4_4$prediction_inner < 0, 0,
Datensatz_4_4$prediction_inner)

Datensatz_4_4$prediction_inner

### MSE berechnen 4###

MSE_DF_4.1 <- data.frame(pred = Datensatz_4_4$prediction_inner,
actual = Datensatz_4_4$Hämoglobin)

MSE_4.1<-mean((MSE_DF_4.1$actual - MSE_DF_4.1$pred)^2)

MSE_4.1

#ohne RE : 1.304894

#mit RE :  1.281395

# out-off sample performance 4 ohne RE###

Gruppe4$prediction_inner <- predict(tobit_4,newdata = Gruppe4)

Gruppe4$prediction_inner

### MSE berechnen 4###

MSE_DF_4.2 <- data.frame(pred = Gruppe4$prediction_inner, actual
= Gruppe4$Hämoglobin)

MSE_4.2<-mean((MSE_DF_4.2$actual - MSE_DF_4.2$pred)^2)

MSE_4.2

#ohne RE : 0.9843887

#mit RE :  0.624709

### Differenz der MSEs 4###

Diff_tobit_4<-MSE_4.2-MSE_4.1

Diff_tobit_4

#ohne RE : -0.320505

#mit RE :  -0.6566857

### Datensatz 5 ###

### fractional polynomials 5 ###
```

```
#df=2 !

model_fractional_5<- mfp(Hämoglobin ~fp(DT_EX1_342.41nm, df = 4, select = 0.05) + fp( AT_EX2_363.92nm, df = 4, select = 0.05) + fp( AR_EX2_363.92nm, df = 4,

                              select = 0.05)+
fp( AR_EX2_557.5nm, df = 4, select = 0.05) +
fp( DT_EX1_586.83nm, df = 4, select = 0.05) , data=Datensatz_4_5)

model_fractional_5

#Datentransformation 5

#Trainingsdaten

Datensatz_4_5$AR_EX2_557.5nm_new <- I(Datensatz_4_5$AR_EX2_557.5nm^1)

Datensatz_4_5$DT_EX1_586.83nm_new <- I(Datensatz_4_5$DT_EX1_586.83nm^-2)+I(Datensatz_4_5$DT_EX1_586.83nm^-2*log(Datensatz_4_5$DT_EX1_586.83nm))

#Testdaten

Gruppe5$AR_EX2_557.5nm_new <-I(Gruppe5$AR_EX2_557.5nm^1)

Gruppe5$DT_EX1_586.83nm_new <-I(Gruppe5$DT_EX1_586.83nm^-2)+I(Gruppe5$DT_EX1_586.83nm^-2*log(Gruppe5$DT_EX1_586.83nm)

tobit_5<- survreg(Surv(Hämoglobin, Hämoglobin>0, type='left') ~ AR_EX2_557.5nm_new + DT_EX1_586.83nm_new

                      ,data=Datensatz_4_5, dist='gaussian')

summary(tobit_5)

step(tobit_5,direction="backward") #AR_EX2_557.5nm_new wird entfernt

tobit_5<- survreg(Surv(Hämoglobin, Hämoglobin>0, type='left') ~ DT_EX1_586.83nm_new

                      ,data=Datensatz_4_5, dist='gaussian')

summary(tobit_5)

# predicted values ohne RE

Datensatz_4_5$prediction_inner <- predict(tobit_5,type="response")

Datensatz_4_5$prediction_inner

Datensatz_4_5$prediction_inner <- ifelse(Datensatz_4_5$prediction_inner < 0, 0, Datensatz_4_5$prediction_inner)
```

```
Datensatz_4_5$prediction_inner

### MSE berechnen 5###

MSE_DF_5.1 <- data.frame(pred = Datensatz_4_5$prediction_inner,
actual = Datensatz_4_5$Hämoglobin)

MSE_5.1<-mean((MSE_DF_5.1$actual - MSE_DF_5.1$pred)^2)

MSE_5.1

#ohne RE : 1.332092

#mit RE  : 1.365008

# out-off sample performance 5 ohne RE###

Gruppe5$prediction_inner <- predict(tobit_5,newdata = Gruppe5)

Gruppe5$prediction_inner

### MSE berechnen 5###

MSE_DF_5.2 <- data.frame(pred = Gruppe5$prediction_inner, actual
= Gruppe5$Hämoglobin)

MSE_5.2<-mean((MSE_DF_5.2$actual - MSE_DF_5.2$pred)^2)

MSE_5.2

#ohne RE : 0.9534782

#mit RE  : 0.2642826

### Differenz der MSEs 5###

Diff_tobit_5<-MSE_5.2-MSE_5.1

Diff_tobit_5

#ohne RE : -0.3786136

#mit RE  : -1.100726


### Datensatz gesamt ###

### fractional polynomials gesamt ###

#df=2 !

model_fractional_gesamt<- mfp(Hämoglobin ~fp(DT_EX1_342.41nm, df
= 4, select = 0.05) + fp( AT_EX2_363.92nm, df = 4, select = 0.05)
+  fp( AR_EX2_363.92nm, df = 4,

                              select = 0.05)+
fp( AR_EX2_557.5nm, df = 4, select = 0.05) +
fp( DT_EX1_586.83nm, df = 4, select = 0.05) , data=Hämoglobin)
```

```
model_fractional_gesamt

Hämoglobin$AT_EX2_363.92nm_new<-
I((Hämoglobin$AT_EX2_363.92nm/10)^1)

Hämoglobin$AR_EX2_557.5nm_new<- I(Hämoglobin$AR_EX2_557.5nm^1)

Hämoglobin$DT_EX1_586.83nm_new<- I(Hämoglobin$DT_EX1_586.83nm^-
2)+I(Hämoglobin$DT_EX1_586.83nm^-
2*log(Hämoglobin$DT_EX1_586.83nm))

tobit_gesamt<- survreg(Surv(Hämoglobin, Hämoglobin>0,
type='left') ~ AT_EX2_363.92nm_new + AR_EX2_557.5nm_new
+DT_EX1_586.83nm_new

                   ,data=Hämoglobin, dist='gaussian')

summary(tobit_gesamt)

step(tobit_gesamt,direction="backward") #AR_EX2_557.5nm_new wird
entfernt

tobit_gesamt<- survreg(Surv(Hämoglobin, Hämoglobin>0,
type='left') ~ AT_EX2_363.92nm_new +DT_EX1_586.83nm_new

                       ,data=Hämoglobin, dist='gaussian')

summary(tobit_gesamt)


# predicted values ohne RE

Hämoglobin$prediction_inner <-
predict(tobit_gesamt,type="response")

Hämoglobin$prediction_inner

Hämoglobin$prediction_inner <-
ifelse(Hämoglobin$prediction_inner < 0, 0,
Hämoglobin$prediction_inner)

Hämoglobin$prediction_inner


### MSE berechnen gesamr###

MSE_DF_gesamt <- data.frame(pred = Hämoglobin$prediction_inner,
actual = Hämoglobin$Hämoglobin)

MSE_gesamt<-mean((MSE_DF_gesamt$actual - MSE_DF_gesamt$pred)^2)

MSE_gesamt

#ohne RE : 1.103596

#mit RE :  1.1032
```

```
library(ggplot2)

ggplot(MSE_DF_gesamt, aes(x = actual, y = pred)) +

  geom_point(alpha = 0.5, color = 'blue') +

  labs(title = 'Actual vs. Predicted Values',

       x = 'Actual Values',

       y = 'Predicted Values') +

  # Calculate and plot the error as vertical lines

  geom_segment(aes(x = actual, xend = actual, y = actual, yend =
pred), color = 'red', linetype = 'dashed') +

  # Set log scale for both x and y axes

  #scale_x_log10() +

  #scale_y_log10() +

  xlim(c(min(MSE_DF_gesamt$actual), max(MSE_DF_gesamt$actual)))
+

  ylim(c(min(MSE_DF_gesamt$actual), max(MSE_DF_gesamt$actual)))
+

  theme_minimal()

### Mittelwert der 5 MSE-Differenzen berechnen ###

Unterschied<-(Diff_tobit_1 + Diff_tobit_2 + Diff_tobit_3 +
Diff_tobit_4 + Diff_tobit_5)/5

Unterschied

#ohne RE : 0.5435825

#mit RE :  0.08389727

MSE_global_corrected<-MSE_gesamt + Unterschied

MSE_global_corrected

#ohne RE : 1.647179

#mit RE :  1.187097

#mit RE ohne predicted values=0 :


var(Hämoglobin$Hämoglobin)

#3.291052
```
