# Supplementary material for: Intelligent surgical drainage - digitizing the analysis of drainage fluid in patients with surgical drains
Source: PLoS One. 2025 Jul 28;20(7):e0325072. doi: 10.1371/journal.pone.0325072 (PMC12303269; doi:10.1371/journal.pone.0325072)
Supplement: S3 File — (PDF) [file pone.0325072.s011.pdf]

```
library(readxl)

library(survival)

library(mfp)

setwd("F:/1. Klinisches Semester/Promotion/R Analyse")

Hämoglobin_binominal_randomized <-read_excel("7_hemoglobin input
dataset for R.xlsx")


# Zahlen als Faktor umdefinieren

Hämoglobin_binominal_randomized$Binär <-
as.factor(Hämoglobin_binominal_randomized$Binär)


# Aufteilung der Daten in Gruppen

set.seed(2023)

sample_size <- nrow(Hämoglobin_binominal_randomized)

set_proportions <- c(Gruppe1 = 0.2, Gruppe2 = 0.2, Gruppe3 = 0.2,
Gruppe4 = 0.2, Gruppe5 = 0.2)

set_frequencies <- diff(floor(sample_size * cumsum(c(0,
set_proportions))))

Hämoglobin_binominal_randomized$set <-
sample(rep(names(set_proportions), times = set_frequencies))


Gruppe1_binominal_randomized <-
Hämoglobin_binominal_randomized[Hämoglobin_binominal_randomized$
set == "Gruppe1", ]

Gruppe2_binominal_randomized <-
Hämoglobin_binominal_randomized[Hämoglobin_binominal_randomized$
set == "Gruppe2", ]

Gruppe3_binominal_randomized <-
Hämoglobin_binominal_randomized[Hämoglobin_binominal_randomized$
set == "Gruppe3", ]

Gruppe4_binominal_randomized <-
Hämoglobin_binominal_randomized[Hämoglobin_binominal_randomized$
set == "Gruppe4", ]

Gruppe5_binominal_randomized <-
Hämoglobin_binominal_randomized[Hämoglobin_binominal_randomized$
set == "Gruppe5", ]
```

```
# Datensätze aus Gruppen bilden

Datensatz_4_1_binominal_randomized <-
rbind(Gruppe2_binominal_randomized, Gruppe3_binominal_randomized,
Gruppe4_binominal_randomized, Gruppe5_binominal_randomized)

Datensatz_4_2_binominal_randomized <-
rbind(Gruppe1_binominal_randomized, Gruppe3_binominal_randomized,
Gruppe4_binominal_randomized, Gruppe5_binominal_randomized)

Datensatz_4_3_binominal_randomized <-
rbind(Gruppe1_binominal_randomized, Gruppe2_binominal_randomized,
Gruppe4_binominal_randomized, Gruppe5_binominal_randomized)

Datensatz_4_4_binominal_randomized <-
rbind(Gruppe1_binominal_randomized, Gruppe2_binominal_randomized,
Gruppe3_binominal_randomized, Gruppe5_binominal_randomized)

Datensatz_4_5_binominal_randomized <-
rbind(Gruppe1_binominal_randomized, Gruppe2_binominal_randomized,
Gruppe3_binominal_randomized, Gruppe4_binominal_randomized)

library(lme4)

# Erstellen des logistischen Regressionsmodells für
Datensatz_4_1

glmerControl(optimizer = "bobyqa", optCtrl = list(maxfun =
100000))

glmerControl(optCtrl = list(absTol = 1e-6, relTol = 1e-6))

glmerControl(optimizer = "bobyqa")


lmer_Hämoglobin_datensatz_4_1_binominal <- glmer(Binär ~
DT_EX1_342.41nm+AT_EX2_363.92nm+AR_EX2_363.92nm+AR_EX2_557.5nm+D
T_EX1_586.83nm  + (1 | Patient),family = "binomial", data =
Datensatz_4_1_binominal_randomized)

summary(lmer_Hämoglobin_datensatz_4_1_binominal)

#install.packages("lmerTest")

#install.packages("MuMIn")

library(lmerTest)

library(MuMIn)

options(na.action = "na.fail")


dredge(lmer_Hämoglobin_datensatz_4_1_binominal , rank = "AIC")
```

```
lmer_Hämoglobin_datensatz_4_1_binominal <- glmer(Binär ~ DT_EX1_342.41nm+AT_EX2_363.92nm+AR_EX2_363.92nm+AR_EX2_557.5nm+DT_EX1_586.83nm  + (1 | Patient)

                                                ,family = "binomial", data = Datensatz_4_1_binominal_randomized , glmerControl(optimizer = "bobyqa"))


summary(lmer_Hämoglobin_datensatz_4_1_binominal)

# Erstellen des Regressionsmodells für Datensatz_4_2

lmer_Hämoglobin_datensatz_4_2_binominal <- glmer(Binär ~ DT_EX1_342.41nm+AT_EX2_363.92nm+AR_EX2_363.92nm+AR_EX2_557.5nm+DT_EX1_586.83nm   + (1 | Patient),family = "binomial",

                                                data = Datensatz_4_2_binominal_randomized , glmerControl(optimizer = "bobyqa"))

summary(lmer_Hämoglobin_datensatz_4_2_binominal)

# best subset selection

dredge(lmer_Hämoglobin_datensatz_4_2_binominal , rank = "AIC")

lmer_Hämoglobin_datensatz_4_2_binominal <- glmer(Binär ~ DT_EX1_342.41nm+AT_EX2_363.92nm+AR_EX2_363.92nm+AR_EX2_557.5nm+DT_EX1_586.83nm   + (1 | Patient),family = "binomial", data = Datensatz_4_2_binominal_randomized , glmerControl(optimizer = "bobyqa"))

summary(lmer_Hämoglobin_datensatz_4_2_binominal)

# Erstellen des logistischen Regressionsmodells für Datensatz_4_3

lmer_Hämoglobin_datensatz_4_3_binominal <- glmer(Binär ~ DT_EX1_342.41nm+AT_EX2_363.92nm+AR_EX2_363.92nm+AR_EX2_557.5nm+DT_EX1_586.83nm  + (1 | Patient),family = "binomial",

                                                data = Datensatz_4_3_binominal_randomized, glmerControl(optimizer = "bobyqa"))


summary(lmer_Hämoglobin_datensatz_4_3_binominal)

# best subset selection

dredge(lmer_Hämoglobin_datensatz_4_3_binominal , rank = "AIC")
```

```
lmer_Hämoglobin_datensatz_4_3_binominal <- glmer(Binär ~ 
DT_EX1_342.41nm+AT_EX2_363.92nm+AR_EX2_363.92nm+AR_EX2_557.5nm+D
T_EX1_586.83nm  +(1 | Patient),family = "binomial",

                                                data = 
Datensatz_4_3_binominal_randomized, glmerControl(optimizer = 
"bobyqa"))

summary(lmer_Hämoglobin_datensatz_4_3_binominal)

# Erstellen des logistischen Regressionsmodells für 
Datensatz_4_4

lmer_Hämoglobin_datensatz_4_4_binominal <- glmer(Binär ~ 
DT_EX1_342.41nm+AT_EX2_363.92nm+AR_EX2_363.92nm+AR_EX2_557.5nm+D
T_EX1_586.83nm + (1 | Patient),family = "binomial",

                                                data = 
Datensatz_4_4_binominal_randomized, glmerControl(optimizer = 
"bobyqa"))

summary(lmer_Hämoglobin_datensatz_4_4_binominal)

# best subset selection

dredge(lmer_Hämoglobin_datensatz_4_4_binominal , rank = "AIC")

lmer_Hämoglobin_datensatz_4_4_binominal <- glmer(Binär ~ 
DT_EX1_342.41nm+AT_EX2_363.92nm+AR_EX2_363.92nm+AR_EX2_557.5nm+D
T_EX1_586.83nm   +(1 | Patient),family = "binomial",

                                                data = 
Datensatz_4_4_binominal_randomized, glmerControl(optimizer = 
"bobyqa"))

summary(lmer_Hämoglobin_datensatz_4_4_binominal)

# Erstellen des logistischen Regressionsmodells für 
Datensatz_4_5

lmer_Hämoglobin_datensatz_4_5_binominal <- glmer(Binär ~ 
DT_EX1_342.41nm+AT_EX2_363.92nm+AR_EX2_363.92nm+AR_EX2_557.5nm+D
T_EX1_586.83nm   + (1 | Patient),family = "binomial",

                                                data = 
Datensatz_4_5_binominal_randomized, glmerControl(optimizer = 
"bobyqa"))

summary(lmer_Hämoglobin_datensatz_4_5_binominal)

# Backwards selection

dredge(lmer_Hämoglobin_datensatz_4_5_binominal , rank = "AIC")

lmer_Hämoglobin_datensatz_4_5_binominal <- glmer(Binär ~ 
DT_EX1_342.41nm+AT_EX2_363.92nm+AR_EX2_363.92nm+AR_EX2_557.5nm+D
T_EX1_586.83nm  +(1 | Patient),family = "binomial",
```

```
                                                data = 
Datensatz_4_5_binominal_randomized, glmerControl(optimizer = 
"bobyqa"))



summary(lmer_Hämoglobin_datensatz_4_5_binominal)


# Erstellen des logistischen Regressionsmodells für den globalen 
Datensatz

lmer_Hämoglobin_binominal_global <- glmer(Binär ~ 
DT_EX1_342.41nm+AT_EX2_363.92nm+AR_EX2_363.92nm+AR_EX2_557.5nm+D
T_EX1_586.83nm   + (1 | Patient),family = "binomial",

                                         data = 
Hämoglobin_binominal_randomized, glmerControl(optimizer = 
"bobyqa"))


summary(lmer_Hämoglobin_binominal_global)

# Best subset selection

dredge(lmer_Hämoglobin_binominal_global , rank = "AIC")

lmer_Hämoglobin_binominal_global <- glmer(Binär ~ 
DT_EX1_342.41nm+AT_EX2_363.92nm+AR_EX2_363.92nm+AR_EX2_557.5nm+D
T_EX1_586.83nm  + (1 | Patient),family = "binomial",

                                         data = 
Hämoglobin_binominal_randomized, glmerControl(optimizer = 
"bobyqa"))


summary(lmer_Hämoglobin_binominal_global)

# inner sample performance


library(caret)

library(ConfusionTableR)

# Datensatz_4_1

Datensatz_4_1_binominal_randomized$prediction_inner <- 
predict(lmer_Hämoglobin_datensatz_4_1_binominal,newdata = 
Datensatz_4_1_binominal_randomized,type = "response")

Datensatz_4_1_binominal_randomized$prediction_inner
```

```
Datensatz_4_1_binominal_randomized$pred_default<-
ifelse(Datensatz_4_1_binominal_randomized$prediction_inner>=0.5,
"1", "0")


Datensatz_4_1_binominal_randomized$pred_default<-
as.factor(Datensatz_4_1_binominal_randomized$pred_default)


xtab_inner_1 <-
table(Datensatz_4_1_binominal_randomized$pred_default,
Datensatz_4_1_binominal_randomized$Binär)

confusionMatrix(xtab_inner_1, positive = "1")

balanced_accuracy_Datensatz_4_1 <-
confusionMatrix(Datensatz_4_1_binominal_randomized$pred_default,
Datensatz_4_1_binominal_randomized$Binär)$byClass["Balanced
Accuracy"]

balanced_accuracy_Datensatz_4_1

#Balanced Accuracy :  0.9101449

# Datensatz_4_2

Datensatz_4_2_binominal_randomized$prediction_inner <-
predict(lmer_Hämoglobin_datensatz_4_2_binominal,newdata =
Datensatz_4_2_binominal_randomized,type = "response")

Datensatz_4_2_binominal_randomized$prediction_inner

Datensatz_4_2_binominal_randomized$pred_default<-
ifelse(Datensatz_4_2_binominal_randomized$prediction_inner>=0.5,
"1", "0")

Datensatz_4_2_binominal_randomized$pred_default<-
as.factor(Datensatz_4_2_binominal_randomized$pred_default)


xtab_inner_2 <-
table(Datensatz_4_2_binominal_randomized$pred_default,
Datensatz_4_2_binominal_randomized$ Binär)

xtab_inner_2

confusionMatrix(xtab_inner_2, positive = "1")

balanced_accuracy_Datensatz_4_2 <-
confusionMatrix(Datensatz_4_2_binominal_randomized$pred_default,
Datensatz_4_2_binominal_randomized$ Binär)$byClass["Balanced
Accuracy"]

balanced_accuracy_Datensatz_4_2
```

```
# Balanced Accuracy : 0.9143929

# Datensatz_4_3

Datensatz_4_3_binominal_randomized$prediction_inner <-
predict(lmer_Hämoglobin_datensatz_4_3_binominal,newdata =
Datensatz_4_3_binominal_randomized,type = "response")

Datensatz_4_3_binominal_randomized$prediction_inner

Datensatz_4_3_binominal_randomized$pred_default<-
ifelse(Datensatz_4_3_binominal_randomized$prediction_inner>=0.5,
"1", "0")

Datensatz_4_3_binominal_randomized$pred_default<-
as.factor(Datensatz_4_3_binominal_randomized$pred_default)


xtab_inner_3 <-
table(Datensatz_4_3_binominal_randomized$pred_default,
Datensatz_4_3_binominal_randomized$ Binär)

xtab_inner_3

confusionMatrix(xtab_inner_3, positive = "1")

balanced_accuracy_Datensatz_4_3 <-
confusionMatrix(Datensatz_4_3_binominal_randomized$pred_default,
Datensatz_4_3_binominal_randomized$ Binär)$byClass["Balanced
Accuracy"]

balanced_accuracy_Datensatz_4_3

# Balanced Accuracy : 0.9067495

# Datensatz_4_4

Datensatz_4_4_binominal_randomized$prediction_inner <-
predict(lmer_Hämoglobin_datensatz_4_4_binominal,newdata =
Datensatz_4_4_binominal_randomized,type = "response")

Datensatz_4_4_binominal_randomized$prediction_inner

Datensatz_4_4_binominal_randomized$pred_default<-
ifelse(Datensatz_4_4_binominal_randomized$prediction_inner>=0.5,
"1", "0")

Datensatz_4_4_binominal_randomized$pred_default<-
as.factor(Datensatz_4_4_binominal_randomized$pred_default)

xtab_inner_4 <-
table(Datensatz_4_4_binominal_randomized$pred_default,
Datensatz_4_4_binominal_randomized$ Binär)

xtab_inner_4
```

```
confusionMatrix(xtab_inner_4, positive = "1")

balanced_accuracy_Datensatz_4_4 <-
confusionMatrix(Datensatz_4_4_binominal_randomized$pred_default,
Datensatz_4_4_binominal_randomized$ Binär)$byClass["Balanced
Accuracy"]

balanced_accuracy_Datensatz_4_4

#Balanced Accuracy : 0.9130435

# Datensatz_4_5

Datensatz_4_5_binominal_randomized$prediction_inner <-
predict(lmer_Hämoglobin_datensatz_4_5_binominal,newdata =
Datensatz_4_5_binominal_randomized,type = "response")

Datensatz_4_5_binominal_randomized$prediction_inner

Datensatz_4_5_binominal_randomized$pred_default<-
ifelse(Datensatz_4_5_binominal_randomized$prediction_inner>=0.5,
"1", "0")

Datensatz_4_5_binominal_randomized$pred_default<-
as.factor(Datensatz_4_5_binominal_randomized$pred_default)

xtab_inner_5 <-
table(Datensatz_4_5_binominal_randomized$pred_default,
Datensatz_4_5_binominal_randomized$ Binär)

xtab_inner_5


confusionMatrix(xtab_inner_5, positive = "1")

balanced_accuracy_Datensatz_4_5 <-
confusionMatrix(Datensatz_4_5_binominal_randomized$pred_default,
Datensatz_4_5_binominal_randomized$ Binär)$byClass["Balanced
Accuracy"]

balanced_accuracy_Datensatz_4_5

#Balanced Accuracy : 0.9307807


# Datensatz_global

Hämoglobin_binominal_randomized$prediction_inner <-
predict(lmer_Hämoglobin_binominal_global,newdata =
Hämoglobin_binominal_randomized,type = "response")

Hämoglobin_binominal_randomized$prediction_inner

Hämoglobin_binominal_randomized$pred_default<-
ifelse(Hämoglobin_binominal_randomized$prediction_inner>=0.5,
"1", "0")
```

```
Hämoglobin_binominal_randomized$pred_default<-
as.factor(Hämoglobin_binominal_randomized$pred_default)

xtab_inner_global <-
table(Hämoglobin_binominal_randomized$pred_default,
Hämoglobin_binominal_randomized$ Binär)

xtab_inner_global


#

#    0   1

#0 124  18

#1  18 265

Hämoglobin_binominal_randomized$pred_default <-
as.factor(Hämoglobin_binominal_randomized$pred_default)

Hämoglobin_binominal_randomized$Binär <-
as.factor(Hämoglobin_binominal_randomized$Binär)

confusionMatrix(xtab_inner_global, positive = "1")

balanced_accuracy_global <-
confusionMatrix(Hämoglobin_binominal_randomized$pred_default,
Hämoglobin_binominal_randomized$ Binär)$byClass["Balanced
Accuracy"]

balanced_accuracy_global

#Balanced Accuracy : 0.9048176


# Modellvalidierung auf ausgelassenem Datensatz

# Datensatz_4_1

# Hier musste ich jeweils all.new.levels = TRUE setzen, sonst
kommt es zu dem Fehler den ich dir gezeigt hatte

Gruppe1_binominal_randomized$prediction <-
predict(lmer_Hämoglobin_datensatz_4_1_binominal,newdata =
Gruppe1_binominal_randomized, allow.new.levels=TRUE, type =
"response")

Gruppe1_binominal_randomized

Gruppe1_binominal_randomized$pred_default <-
ifelse(Gruppe1_binominal_randomized$prediction>=0.5, "1", "0")

Gruppe1_binominal_randomized$pred_default<-
as.factor(Gruppe1_binominal_randomized$pred_default)
```

```
xtab1 <- table(Gruppe1_binominal_randomized$pred_default,
Gruppe1_binominal_randomized$ Binär)

xtab1

#   0  1

#0 71  5

#1  1 13


confusionMatrix(xtab1, positive = "1")

balanced_accuracy_Gruppe1 <-
confusionMatrix(Gruppe1_binominal_randomized$pred_default,
Gruppe1_binominal_randomized$ Binär)$byClass["Balanced Accuracy"]

balanced_accuracy_Gruppe1

#Balanced Accuracy : 0.8457854

# Datensatz_4_2

Gruppe2_binominal_randomized$prediction <-
predict(lmer_Hämoglobin_datensatz_4_2_binominal,newdata =
Gruppe2_binominal_randomized,allow.new.levels=TRUE, type =
"response")

Gruppe2_binominal_randomized

Gruppe2_binominal_randomized$pred_default <-
ifelse(Gruppe2_binominal_randomized$prediction>=0.5, "1", "0")

Gruppe2_binominal_randomized$pred_default<-
as.factor(Gruppe2_binominal_randomized$pred_default)

xtab2 <- table(Gruppe2_binominal_randomized$pred_default,
Gruppe2_binominal_randomized$ Binär)

xtab2

#   0  1

#0 64 10

#1  3 14

confusionMatrix(xtab2, positive = "1")

balanced_accuracy_Gruppe2 <-
confusionMatrix(Gruppe2_binominal_randomized$pred_default,
Gruppe2_binominal_randomized$ Binär)$byClass["Balanced Accuracy"]

balanced_accuracy_Gruppe2

# Balanced Accuracy :   0.9064685
```

```
# Datensatz_4_3

Gruppe3_binominal_randomized$prediction <-
predict(lmer_Hämoglobin_datensatz_4_3_binominal,newdata =
Gruppe3_binominal_randomized, allow.new.levels=TRUE,type =
"response")

Gruppe3_binominal_randomized


Gruppe3_binominal_randomized$pred_default <-
ifelse(Gruppe3_binominal_randomized$prediction>=0.5, "1", "0")

Gruppe3_binominal_randomized$pred_default<-
as.factor(Gruppe3_binominal_randomized$pred_default)

xtab3 <- table(Gruppe3_binominal_randomized$pred_default,
Gruppe3_binominal_randomized$ Binär)

xtab3

#   0  1

#0 72  2

#1  4 12

confusionMatrix(xtab3, positive = "1")

balanced_accuracy_Gruppe3 <-
confusionMatrix(Gruppe3_binominal_randomized$pred_default,
Gruppe3_binominal_randomized$ Binär)$byClass["Balanced Accuracy"]

balanced_accuracy_Gruppe3

# Balanced Accuracy : 0.8383333

# Datensatz_4_4

Gruppe4_binominal_randomized$prediction <-
predict(lmer_Hämoglobin_datensatz_4_4_binominal,newdata =
Gruppe4_binominal_randomized, allow.new.levels=TRUE, type =
"response")

Gruppe4_binominal_randomized

Gruppe4_binominal_randomized$pred_default <-
ifelse(Gruppe4_binominal_randomized$prediction>=0.5, "1", "0")

Gruppe4_binominal_randomized$pred_default<-
as.factor(Gruppe4_binominal_randomized$pred_default)

xtab4 <- table(Gruppe4_binominal_randomized$pred_default,
Gruppe4_binominal_randomized$ Binär)

xtab4
```

```
#   0  1

#0 71  7

#1  1 12

confusionMatrix(xtab4, positive = "1")

balanced_accuracy_Gruppe4 <-
confusionMatrix(Gruppe4_binominal_randomized$pred_default,
Gruppe4_binominal_randomized$ Binär)$byClass["Balanced Accuracy"]

balanced_accuracy_Gruppe4

# Balanced Accuracy : 0.8685142

# Datensatz_4_5

Gruppe5_binominal_randomized$prediction <-
predict(lmer_Hämoglobin_datensatz_4_5_binominal,newdata =
Gruppe5_binominal_randomized, allow.new.levels=TRUE, type =
"response")

Gruppe5_binominal_randomized

Gruppe5_binominal_randomized$pred_default <-
ifelse(Gruppe5_binominal_randomized$prediction>=0.5, "1", "0")

Gruppe5_binominal_randomized$pred_default<-
as.factor(Gruppe5_binominal_randomized$pred_default)

xtab5 <- table(Gruppe5_binominal_randomized$pred_default,
Gruppe5_binominal_randomized$ Binär)

xtab5

#   0  1

#0 69  9

#1  4  9

confusionMatrix(xtab5, positive = "1")

balanced_accuracy_Gruppe5 <-
confusionMatrix(Gruppe5_binominal_randomized$pred_default,
Gruppe5_binominal_randomized$ Binär)$byClass["Balanced Accuracy"]

balanced_accuracy_Gruppe5

# Balanced Accuracy :  0.8566667

#Mittelwert der balanced accuracys und Vergleich mit globalem
Modell

Diff_1 = balanced_accuracy_Datensatz_4_1   -
balanced_accuracy_Gruppe1

Diff_1
```

```
Diff_2 = balanced_accuracy_Datensatz_4_2  -
balanced_accuracy_Gruppe2

Diff_2

Diff_3 = balanced_accuracy_Datensatz_4_3 -
balanced_accuracy_Gruppe3

Diff_3

Diff_4 = balanced_accuracy_Datensatz_4_4 -
balanced_accuracy_Gruppe4

Diff_4

Diff_5 = balanced_accuracy_Datensatz_4_5  -
balanced_accuracy_Gruppe5

Diff_5


Diff_data_bal_acc <- c(Diff_1, Diff_2, Diff_3, Diff_4, Diff_5)

mean_bal_acc <- mean(Diff_data_bal_acc)

mean_bal_acc

sd_bal_acc <- sd(Diff_data_bal_acc)

sd_bal_acc

var_bal_acc <- var(Diff_data_bal_acc)

var_bal_acc


# Mittelwert_bal_acc =  0.1143801

# Standardabweichung_bal_acc = 0.06973908

# Varianz_bal_acc = 0.00486354


# balanced accuracy global - Mittelwert_bal_acc


Bal_acc_global_bereinigt <- balanced_accuracy_global  -
mean(Diff_data_bal_acc)

Bal_acc_global_bereinigt


# Bal. acc. globales Modell - Mittelwert_bal_acc = 0.8529489

# inner sample performance AUC
```

```
#install.packages("ROCR")

library(ROCR)


# Datensatz_4_1



pred <-
prediction(Datensatz_4_1_binominal_randomized$prediction_inner,
Datensatz_4_1_binominal_randomized$ Binär)

perf <- performance(pred, "tpr", "fpr")

plot(perf, colorize=TRUE)

Datensatz_4_1_AUC<- unlist(slot(performance(pred, "auc"),
"y.values"))

Datensatz_4_1_AUC

# AUC =  0.9761932

# Datensatz_4_2

pred <-
prediction(Datensatz_4_2_binominal_randomized$prediction_inner,
Datensatz_4_2_binominal_randomized$ Binär)

perf <- performance(pred, "tpr", "fpr")

plot(perf, colorize=TRUE)

Datensatz_4_2_AUC <- unlist(slot(performance(pred, "auc"),
"y.values"))

Datensatz_4_2_AUC

# AUC =   0.9801422

# Datensatz_4_3

pred <-
prediction(Datensatz_4_3_binominal_randomized$prediction_inner,
Datensatz_4_3_binominal_randomized$ Binär)

perf <- performance(pred, "tpr", "fpr")

plot(perf, colorize=TRUE)

Datensatz_4_3_AUC <-unlist(slot(performance(pred, "auc"),
"y.values"))

Datensatz_4_3_AUC

# AUC = 0.9789966
```

```
# Datensatz_4_4

pred <-
prediction(Datensatz_4_4_binominal_randomized$prediction_inner,
Datensatz_4_4_binominal_randomized$ Binär)

perf <- performance(pred, "tpr", "fpr")

plot(perf, colorize=TRUE)

Datensatz_4_4_AUC <- unlist(slot(performance(pred, "auc"),
"y.values"))

Datensatz_4_4_AUC


# AUC = 0.975336

# Datensatz_4_5

pred <-
prediction(Datensatz_4_5_binominal_randomized$prediction_inner,
Datensatz_4_5_binominal_randomized$ Binär)

perf <- performance(pred, "tpr", "fpr")

plot(perf, colorize=TRUE)

Datensatz_4_5_AUC <- unlist(slot(performance(pred, "auc"),
"y.values"))

Datensatz_4_5_AUC

# AUC = 0.9850906

# Datensatz_global

pred <-
prediction(Hämoglobin_binominal_randomized$prediction_inner,
Hämoglobin_binominal_randomized$Binär)

perf <- performance(pred, "tpr", "fpr")

plot(perf, colorize=TRUE)

Datensatz_global_AUC <- unlist(slot(performance(pred, "auc"),
"y.values"))

Datensatz_global_AUC

# AUC = 0.9768078

# out-off sample performance AUC

# Datensatz_4_1

pred <- prediction(Gruppe1_binominal_randomized$prediction,
Gruppe1_binominal_randomized$ Binär)
```

```
perf <- performance(pred, "tpr", "fpr")

plot(perf, colorize=TRUE)

Gruppe1_AUC <- unlist(slot(performance(pred, "auc"), "y.values"))

Gruppe1_AUC

# AUC = 0.9412516

# Datensatz_4_2

pred <- prediction(Gruppe2_binominal_randomized$prediction,
Gruppe2_binominal_randomized$ Binär)

perf <- performance(pred, "tpr", "fpr")

plot(perf, colorize=TRUE)


Gruppe2_AUC <- unlist(slot(performance(pred, "auc"), "y.values"))

Gruppe2_AUC

# AUC = 0.9638695

# Datensatz_4_3

pred <- prediction(Gruppe3_binominal_randomized$prediction,
Gruppe3_binominal_randomized$ Binär)

perf <- performance(pred, "tpr", "fpr")

plot(perf, colorize=TRUE)

Gruppe3_AUC <- unlist(slot(performance(pred, "auc"), "y.values"))

Gruppe3_AUC

# AUC = 0.9553333

# Datensatz_4_4

pred <- prediction(Gruppe4_binominal_randomized$prediction,
Gruppe4_binominal_randomized$ Binär)

perf <- performance(pred, "tpr", "fpr")

plot(perf, colorize=TRUE)

Gruppe4_AUC <- unlist(slot(performance(pred, "auc"), "y.values"))

Gruppe4_AUC

# AUC = 0.9705189

# Datensatz_4_5

pred <- prediction(Gruppe5_binominal_randomized$prediction,
Gruppe5_binominal_randomized$ Binär)
```

```
perf <- performance(pred, "tpr", "fpr")
plot(perf, colorize=TRUE)

Gruppe5_AUC <- unlist(slot(performance(pred, "auc"), "y.values"))
Gruppe5_AUC
# AUC = 0.9173333




#Mittelwert der AUC und Vergleich mit globalem Model
Diff_1_AUC = Datensatz_4_1_AUC   - Gruppe1_AUC
Diff_1_AUC
Diff_2_AUC = Datensatz_4_2_AUC  - Gruppe2_AUC
Diff_2_AUC
Diff_3_AUC = Datensatz_4_3_AUC - Gruppe3_AUC
Diff_3_AUC
Diff_4_AUC = Datensatz_4_4_AUC -  Gruppe4_AUC
Diff_4_AUC
Diff_5_AUC = Datensatz_4_5_AUC  - Gruppe5_AUC
Diff_5_AUC

Diff_data_AUC <- c(Diff_1_AUC, Diff_2_AUC, Diff_3_AUC,
Diff_4_AUC, Diff_5_AUC)
mean_AUC <- mean(Diff_data_AUC)
mean_AUC
sd_AUC <- sd(Diff_data_AUC)
sd_AUC
var_AUC <- var(Diff_data_AUC)
var_AUC

# Mittelwert_AUC = 0.02949041
# Standardabweichung_AUC = 0.02403907
```

```
# Varianz_AUC = 0.0005778767
# AUC global - Mittelwert_AUC
AUC_global_bereinigt<- Datensatz_global_AUC - mean_AUC
AUC_global_bereinigt

# AUC_global - Mittelwert_bal_acc = 0.9473174
```
