# Supplementary material for: Intelligent surgical drainage - digitizing the analysis of drainage fluid in patients with surgical drains
Source: PLoS One. 2025 Jul 28;20(7):e0325072. doi: 10.1371/journal.pone.0325072 (PMC12303269; doi:10.1371/journal.pone.0325072)
Supplement: S4 File — (PDF) [file pone.0325072.s012.pdf]

```r
library(readxl)

library(survival)

library(mfp)

setwd("F:/1. Klinisches Semester/Promotion/R Analyse")

Hämoglobin_binominal_randomized <- read_excel("7_hemoglobin
input dataset for R.xlsx")

# Zahlen als Faktor umdefinieren

Hämoglobin_binominal_randomized$Binär <-
as.factor(Hämoglobin_binominal_randomized$Binär)

# Aufteilung der Daten in Gruppen

set.seed(2023)

sample_size <- nrow(Hämoglobin_binominal_randomized)

set_proportions <- c(Gruppe1 = 0.2, Gruppe2 = 0.2, Gruppe3 = 0.2,
Gruppe4 = 0.2, Gruppe5 = 0.2)

set_frequencies <- diff(floor(sample_size * cumsum(c(0,
set_proportions))))

Hämoglobin_binominal_randomized$set <-
sample(rep(names(set_proportions), times = set_frequencies))


Gruppe1_binominal_randomized <-
Hämoglobin_binominal_randomized[Hämoglobin_binominal_randomized$
set == "Gruppe1", ]

Gruppe2_binominal_randomized <-
Hämoglobin_binominal_randomized[Hämoglobin_binominal_randomized$
set == "Gruppe2", ]

Gruppe3_binominal_randomized <-
Hämoglobin_binominal_randomized[Hämoglobin_binominal_randomized$
set == "Gruppe3", ]

Gruppe4_binominal_randomized <-
Hämoglobin_binominal_randomized[Hämoglobin_binominal_randomized$
set == "Gruppe4", ]

Gruppe5_binominal_randomized <-
Hämoglobin_binominal_randomized[Hämoglobin_binominal_randomized$
set == "Gruppe5", ]



# Datensätze aus Gruppen bilden
```

```
Datensatz_4_1_binominal_randomized <-
rbind(Gruppe2_binominal_randomized, Gruppe3_binominal_randomized,
Gruppe4_binominal_randomized, Gruppe5_binominal_randomized)

Datensatz_4_2_binominal_randomized <-
rbind(Gruppe1_binominal_randomized, Gruppe3_binominal_randomized,
Gruppe4_binominal_randomized, Gruppe5_binominal_randomized)

Datensatz_4_3_binominal_randomized <-
rbind(Gruppe1_binominal_randomized, Gruppe2_binominal_randomized,
Gruppe4_binominal_randomized, Gruppe5_binominal_randomized)

Datensatz_4_4_binominal_randomized <-
rbind(Gruppe1_binominal_randomized, Gruppe2_binominal_randomized,
Gruppe3_binominal_randomized, Gruppe5_binominal_randomized)

Datensatz_4_5_binominal_randomized <-
rbind(Gruppe1_binominal_randomized, Gruppe2_binominal_randomized,
Gruppe3_binominal_randomized, Gruppe4_binominal_randomized)

library(glmtoolbox)

# Erstellen des logistischen Regressionsmodells für
Datensatz_4_1

glm_Hämoglobin_datensatz_4_1_binominal <-
glm(Binär~DT_EX1_342.41nm+AT_EX2_363.92nm+AR_EX2_363.92nm+AR_EX2
_557.5nm+DT_EX1_586.83nm , data =
Datensatz_4_1_binominal_randomized, family = binomial)

summary(glm_Hämoglobin_datensatz_4_1_binominal)

library(lmerTest)

library(MuMIn)


options(na.action = "na.fail")


dredge(glm_Hämoglobin_datensatz_4_1_binominal , rank = "AIC")

glm_Hämoglobin_datensatz_4_1_binominal <- glm(Binär ~
DT_EX1_342.41nm+AT_EX2_363.92nm+AR_EX2_363.92nm+AR_EX2_557.5nm+D
T_EX1_586.83nm

                                                ,family =
"binomial", data = Datensatz_4_1_binominal_randomized)


summary(glm_Hämoglobin_datensatz_4_1_binominal)
```

```
# Erstellen des logistischen Regressionsmodells für Datensatz_4_2

glm_Hämoglobin_datensatz_4_2_binominal <- glm(Binär~DT_EX1_342.41nm+AT_EX2_363.92nm+AR_EX2_363.92nm+AR_EX2_557.5nm+DT_EX1_586.83nm , data = Datensatz_4_2_binominal_randomized, family = binomial)


summary(glm_Hämoglobin_datensatz_4_2_binominal)

# best subset selection

dredge(glm_Hämoglobin_datensatz_4_2_binominal , rank = "AIC")

glm_Hämoglobin_datensatz_4_2_binominal <- glm(Binär ~ DT_EX1_342.41nm+AT_EX2_363.92nm+AR_EX2_363.92nm+AR_EX2_557.5nm+DT_EX1_586.83nm

                                              ,family = "binomial", data = Datensatz_4_2_binominal_randomized)


summary(glm_Hämoglobin_datensatz_4_2_binominal)


# Erstellen des logistischen Regressionsmodells für Datensatz_4_3


glm_Hämoglobin_datensatz_4_3_binominal <- glm(Binär~DT_EX1_342.41nm+AT_EX2_363.92nm+AR_EX2_363.92nm+AR_EX2_557.5nm+DT_EX1_586.83nm , data = Datensatz_4_3_binominal_randomized, family = binomial)

summary(glm_Hämoglobin_datensatz_4_3_binominal)


# best subset selection

dredge(glm_Hämoglobin_datensatz_4_3_binominal , rank = "AIC")

glm_Hämoglobin_datensatz_4_3_binominal <- glm(Binär ~ DT_EX1_342.41nm+AT_EX2_363.92nm+AR_EX2_363.92nm+AR_EX2_557.5nm+DT_EX1_586.83nm

                                              ,family = "binomial", data = Datensatz_4_3_binominal_randomized)


summary(glm_Hämoglobin_datensatz_4_3_binominal)
```

```
# Erstellen des logistischen Regressionsmodells für
Datensatz_4_4

glm_Hämoglobin_datensatz_4_4_binominal <-
glm(Binär~DT_EX1_342.41nm+AT_EX2_363.92nm+AR_EX2_363.92nm+AR_EX2
_557.5nm+DT_EX1_586.83nm , data =
Datensatz_4_4_binominal_randomized, family = binomial)

summary(glm_Hämoglobin_datensatz_4_4_binominal)

# best subset selection

dredge(glm_Hämoglobin_datensatz_4_4_binominal , rank = "AIC")

glm_Hämoglobin_datensatz_4_4_binominal <- glm(Binär ~
DT_EX1_342.41nm+AT_EX2_363.92nm+AR_EX2_363.92nm+AR_EX2_557.5nm+D
T_EX1_586.83nm

                                             ,family =
"binomial", data = Datensatz_4_4_binominal_randomized)

summary(glm_Hämoglobin_datensatz_4_4_binominal)


# Erstellen des logistischen Regressionsmodells für
Datensatz_4_5

glm_Hämoglobin_datensatz_4_5_binominal <-
glm(Binär~DT_EX1_342.41nm+AT_EX2_363.92nm+AR_EX2_363.92nm+AR_EX2
_557.5nm+DT_EX1_586.83nm , data =
Datensatz_4_5_binominal_randomized, family = binomial)


summary(glm_Hämoglobin_datensatz_4_5_binominal)


# Best subset selection

dredge(glm_Hämoglobin_datensatz_4_5_binominal , rank = "AIC")

glm_Hämoglobin_datensatz_4_5_binominal <- glm(Binär ~
DT_EX1_342.41nm+AT_EX2_363.92nm+AR_EX2_363.92nm+AR_EX2_557.5nm+D
T_EX1_586.83nm

                                             ,family =
"binomial", data = Datensatz_4_5_binominal_randomized)
```

```
summary(glm_Hämoglobin_datensatz_4_5_binominal)

# Erstellen des logistischen Regressionsmodells für den globalen Datensatz

glm_Hämoglobin_global_binominal <- glm(Binär~DT_EX1_342.41nm+AT_EX2_363.92nm+AR_EX2_363.92nm+AR_EX2_557.5nm+DT_EX1_586.83nm , data = Hämoglobin_binominal_randomized, family = binomial)


summary(glm_Hämoglobin_global_binominal)

# Best subset selection

dredge(glm_Hämoglobin_global_binominal , rank = "AIC")

glm_Hämoglobin_global_binominal <- glm(Binär ~ DT_EX1_342.41nm+AT_EX2_363.92nm+AR_EX2_363.92nm+AR_EX2_557.5nm+DT_EX1_586.83nm

                                             ,family = "binomial", data = Hämoglobin_binominal_randomized )

summary(glm_Hämoglobin_global_binominal)


# inner sample performance

library(caret)

library(ConfusionTableR)

# Datensatz_4_1

Datensatz_4_1_binominal_randomized$prediction_inner <- predict(glm_Hämoglobin_datensatz_4_1_binominal,newdata = Datensatz_4_1_binominal_randomized,type = "response")

Datensatz_4_1_binominal_randomized$prediction_inner


Datensatz_4_1_binominal_randomized$pred_default<- ifelse(Datensatz_4_1_binominal_randomized$prediction_inner>=0.5, "1", "0")


Datensatz_4_1_binominal_randomized$pred_default<- as.factor(Datensatz_4_1_binominal_randomized$pred_default)

xtab_inner_1 <- table(Datensatz_4_1_binominal_randomized$pred_default, Datensatz_4_1_binominal_randomized$Binär)
```

```
confusionMatrix(xtab_inner_1, positive = "1")

balanced_accuracy_Datensatz_4_1 <-
confusionMatrix(Datensatz_4_1_binominal_randomized$pred_default,
Datensatz_4_1_binominal_randomized$Binär)$byClass["Balanced
Accuracy"]

balanced_accuracy_Datensatz_4_1


#Balanced Accuracy : 0.8859903


# Datensatz_4_2

Datensatz_4_2_binominal_randomized$prediction_inner <-
predict(glm_Hämoglobin_datensatz_4_2_binominal,newdata =
Datensatz_4_2_binominal_randomized,type = "response")

Datensatz_4_2_binominal_randomized$prediction_inner

Datensatz_4_2_binominal_randomized$pred_default<-
ifelse(Datensatz_4_2_binominal_randomized$prediction_inner>=0.5,
"1", "0")

Datensatz_4_2_binominal_randomized$pred_default<-
as.factor(Datensatz_4_2_binominal_randomized$pred_default)

xtab_inner_2 <-
table(Datensatz_4_2_binominal_randomized$pred_default,
Datensatz_4_2_binominal_randomized$Binär)

xtab_inner_2

confusionMatrix(xtab_inner_2, positive = "1")

balanced_accuracy_Datensatz_4_2 <-
confusionMatrix(Datensatz_4_2_binominal_randomized$pred_default,
Datensatz_4_2_binominal_randomized$Binär)$byClass["Balanced
Accuracy"]

balanced_accuracy_Datensatz_4_2

# Balanced Accuracy :  0.8784702


# Datensatz_4_3

Datensatz_4_3_binominal_randomized$prediction_inner <-
predict(glm_Hämoglobin_datensatz_4_3_binominal,newdata =
Datensatz_4_3_binominal_randomized,type = "response")

Datensatz_4_3_binominal_randomized$prediction_inner
```

```
Datensatz_4_3_binominal_randomized$pred_default<-
ifelse(Datensatz_4_3_binominal_randomized$prediction_inner>=0.5,
"1", "0")

Datensatz_4_3_binominal_randomized$pred_default<-
as.factor(Datensatz_4_3_binominal_randomized$pred_default)

xtab_inner_3 <-
table(Datensatz_4_3_binominal_randomized$pred_default,
Datensatz_4_3_binominal_randomized$Binär)

xtab_inner_3

confusionMatrix(xtab_inner_3, positive = "1")

balanced_accuracy_Datensatz_4_3 <-
confusionMatrix(Datensatz_4_3_binominal_randomized$pred_default,
Datensatz_4_3_binominal_randomized$Binär)$byClass["Balanced
Accuracy"]

balanced_accuracy_Datensatz_4_3

# Balanced Accuracy :  0.8892338


# Datensatz_4_4

Datensatz_4_4_binominal_randomized$prediction_inner <-
predict(glm_Hämoglobin_datensatz_4_4_binominal,newdata =
Datensatz_4_4_binominal_randomized,type = "response")

Datensatz_4_4_binominal_randomized$prediction_inner

Datensatz_4_4_binominal_randomized$pred_default<-
ifelse(Datensatz_4_4_binominal_randomized$prediction_inner>=0.5,
"1", "0")

Datensatz_4_4_binominal_randomized$pred_default<-
as.factor(Datensatz_4_4_binominal_randomized$pred_default)

xtab_inner_4 <-
table(Datensatz_4_4_binominal_randomized$pred_default,
Datensatz_4_4_binominal_randomized$Binär)

xtab_inner_4

confusionMatrix(xtab_inner_4, positive = "1")

balanced_accuracy_Datensatz_4_4 <-
confusionMatrix(Datensatz_4_4_binominal_randomized$pred_default,
Datensatz_4_4_binominal_randomized$Binär)$byClass["Balanced
Accuracy"]

balanced_accuracy_Datensatz_4_4

#Balanced Accuracy : 0.8772727
```

```
# Datensatz_4_5

Datensatz_4_5_binominal_randomized$prediction_inner <-
predict(glm_Hämoglobin_datensatz_4_5_binominal,newdata =
Datensatz_4_5_binominal_randomized,type = "response")

Datensatz_4_5_binominal_randomized$prediction_inner

Datensatz_4_5_binominal_randomized$pred_default<-
ifelse(Datensatz_4_5_binominal_randomized$prediction_inner>=0.5,
"1", "0")

Datensatz_4_5_binominal_randomized$pred_default<-
as.factor(Datensatz_4_5_binominal_randomized$pred_default)

xtab_inner_5 <-
table(Datensatz_4_5_binominal_randomized$pred_default,
Datensatz_4_5_binominal_randomized$Binär)

xtab_inner_5


confusionMatrix(xtab_inner_5, positive = "1")

balanced_accuracy_Datensatz_4_5 <-
confusionMatrix(Datensatz_4_5_binominal_randomized$pred_default,
Datensatz_4_5_binominal_randomized$Binär)$byClass["Balanced
Accuracy"]

balanced_accuracy_Datensatz_4_5

#Balanced Accuracy :   0.8957495


# Datensatz_global

Hämoglobin_binominal_randomized$prediction_inner <-
predict(glm_Hämoglobin_global_binominal,newdata =
Hämoglobin_binominal_randomized,type = "response")

Hämoglobin_binominal_randomized$prediction_inner

Hämoglobin_binominal_randomized$pred_default<-
ifelse(Hämoglobin_binominal_randomized$prediction_inner>=0.5,
"1", "0")

Hämoglobin_binominal_randomized$pred_default<-
as.factor(Hämoglobin_binominal_randomized$pred_default)

xtab_inner_global <-
table(Hämoglobin_binominal_randomized$pred_default,
Hämoglobin_binominal_randomized$Binär)

xtab_inner_global
```

```
#0   1

#0 122  22

#1  20 261

confusionMatrix(xtab_inner_global, positive = "1")

balanced_accuracy_global <-
confusionMatrix(Hämoglobin_binominal_randomized$pred_default,
Hämoglobin_binominal_randomized$Binär)$byClass["Balanced
Accuracy"]

balanced_accuracy_global

#Balanced Accuracy : 0.8907082

# Modellvalidierung auf ausgelassenem Datensatz

# Gruppe_1



Gruppe1_binominal_randomized$prediction <-
predict(glm_Hämoglobin_datensatz_4_1_binominal,newdata =
Gruppe1_binominal_randomized,type = "response")

Gruppe1_binominal_randomized

Gruppe1_binominal_randomized$pred_default <-
ifelse(Gruppe1_binominal_randomized$prediction>=0.5, "1", "0")

Gruppe1_binominal_randomized$pred_default<-
as.factor(Gruppe1_binominal_randomized$pred_default)

xtab1 <- table(Gruppe1_binominal_randomized$pred_default,
Gruppe1_binominal_randomized$Binär)

xtab1

confusionMatrix(xtab1, positive = "1")

balanced_accuracy_Gruppe1 <-
confusionMatrix(Gruppe1_binominal_randomized$pred_default,
Gruppe1_binominal_randomized$Binär)$byClass["Balanced Accuracy"]

balanced_accuracy_Gruppe1

#Balanced Accuracy : 0.8815453


# Gruppe_2

Gruppe2_binominal_randomized$prediction <-
predict(glm_Hämoglobin_datensatz_4_2_binominal,newdata =
Gruppe2_binominal_randomized,type = "response")
```

```
Gruppe2_binominal_randomized

Gruppe2_binominal_randomized$pred_default <- 
ifelse(Gruppe2_binominal_randomized$prediction>=0.5, "1", "0")

Gruppe2_binominal_randomized$pred_default<- 
as.factor(Gruppe2_binominal_randomized$pred_default)

xtab2 <- table(Gruppe2_binominal_randomized$pred_default, 
Gruppe2_binominal_randomized$Binär)

xtab2

confusionMatrix(xtab2, positive = "1")

balanced_accuracy_Gruppe2 <- 
confusionMatrix(Gruppe2_binominal_randomized$pred_default, 
Gruppe2_binominal_randomized$Binär)$byClass["Balanced Accuracy"]

balanced_accuracy_Gruppe2


# Balanced Accuracy :  0.92162

# Gruppe_3

Gruppe3_binominal_randomized$prediction <- 
predict(glm_Hämoglobin_datensatz_4_3_binominal, newdata = 
Gruppe3_binominal_randomized,type = "response")

Gruppe3_binominal_randomized

Gruppe3_binominal_randomized$pred_default <- 
ifelse(Gruppe3_binominal_randomized$prediction>=0.5, "1", "0")

Gruppe3_binominal_randomized$pred_default<- 
as.factor(Gruppe3_binominal_randomized$pred_default)

xtab3 <- table(Gruppe3_binominal_randomized$pred_default, 
Gruppe3_binominal_randomized$Binär)

xtab3

confusionMatrix(xtab3, positive = "1")

balanced_accuracy_Gruppe3 <- 
confusionMatrix(Gruppe3_binominal_randomized$pred_default, 
Gruppe3_binominal_randomized$Binär)$byClass["Balanced Accuracy"]

balanced_accuracy_Gruppe3

# Balanced Accuracy : 0.8583333


# Gruppe_4
```

```
Gruppe4_binominal_randomized$prediction <-
predict(glm_Hämoglobin_datensatz_4_4_binominal, newdata =
Gruppe4_binominal_randomized,type = "response")

Gruppe4_binominal_randomized

Gruppe4_binominal_randomized$pred_default <-
ifelse(Gruppe4_binominal_randomized$prediction>=0.5, "1", "0")

Gruppe4_binominal_randomized$pred_default<-
as.factor(Gruppe4_binominal_randomized$pred_default)

xtab4 <- table(Gruppe4_binominal_randomized$pred_default,
Gruppe4_binominal_randomized$Binär)

xtab4

confusionMatrix(xtab4, positive = "1")

balanced_accuracy_Gruppe4 <-
confusionMatrix(Gruppe4_binominal_randomized$pred_default,
Gruppe4_binominal_randomized$Binär)$byClass["Balanced Accuracy"]

balanced_accuracy_Gruppe4

# Balanced Accuracy :  0.8935731


# Gruppe_5

Gruppe5_binominal_randomized$prediction <-
predict(glm_Hämoglobin_datensatz_4_5_binominal, newdata =
Gruppe5_binominal_randomized,type = "response")

Gruppe5_binominal_randomized

Gruppe5_binominal_randomized$pred_default <-
ifelse(Gruppe5_binominal_randomized$prediction>=0.5, "1", "0")

Gruppe5_binominal_randomized$pred_default<-
as.factor(Gruppe5_binominal_randomized$pred_default)

xtab5 <- table(Gruppe5_binominal_randomized$pred_default,
Gruppe5_binominal_randomized$Binär)

xtab5

confusionMatrix(xtab5, positive = "1")

balanced_accuracy_Gruppe5 <-
confusionMatrix(Gruppe5_binominal_randomized$pred_default,
Gruppe5_binominal_randomized$Binär)$byClass["Balanced Accuracy"]

balanced_accuracy_Gruppe5

# Balanced Accuracy : 0.865
```

```
#Mittelwert der balanced accuracys und Vergleich mit globalem Modell

Diff_1 = balanced_accuracy_Datensatz_4_1   - balanced_accuracy_Gruppe1

Diff_1

Diff_2 = balanced_accuracy_Datensatz_4_2  - balanced_accuracy_Gruppe2

Diff_2

Diff_3 = balanced_accuracy_Datensatz_4_3 - balanced_accuracy_Gruppe3

Diff_3

Diff_4 = balanced_accuracy_Datensatz_4_4 - balanced_accuracy_Gruppe4

Diff_4

Diff_5 = balanced_accuracy_Datensatz_4_5  - balanced_accuracy_Gruppe5

Diff_5

Diff_data_bal_acc <- c(Diff_1, Diff_2, Diff_3, Diff_4, Diff_5)

mean_bal_acc <- mean(Diff_data_bal_acc)

mean_bal_acc

sd_bal_acc <- sd(Diff_data_bal_acc)

sd_bal_acc

var_bal_acc <- var(Diff_data_bal_acc)

var_bal_acc


# Mittelwert_bal_acc =  0.001328943

# Standardabweichung_bal_acc = 0.03177623

# Varianz_bal_acc = 0.001009729


# balanced accuracy global - Mittelwert_bal_acc


Bal_acc_global_bereinigt <- balanced_accuracy_global  - mean(Diff_data_bal_acc)

Bal_acc_global_bereinigt
```

```
# Bal. acc. globales Modell - Mittelwert_bal_acc = 0.8893793

# inner sample performance AUC

library(ROCR)

# Datensatz_4_1

pred <-
prediction(Datensatz_4_1_binominal_randomized$prediction_inner,
Datensatz_4_1_binominal_randomized$Binär)

perf <- performance(pred, "tpr", "fpr")

plot(perf, colorize=TRUE)

Datensatz_4_1_AUC<- unlist(slot(performance(pred, "auc"),
"y.values"))

Datensatz_4_1_AUC

# AUC = 0.9543188

# Datensatz_4_2

pred <-
prediction(Datensatz_4_2_binominal_randomized$prediction_inner,
Datensatz_4_2_binominal_randomized$Binär)

perf <- performance(pred, "tpr", "fpr")

plot(perf, colorize=TRUE)

Datensatz_4_2_AUC <- unlist(slot(performance(pred, "auc"),
"y.values"))

Datensatz_4_2_AUC

# AUC =  0.9475754

# Datensatz_4_3

pred <-
prediction(Datensatz_4_3_binominal_randomized$prediction_inner,
Datensatz_4_3_binominal_randomized$Binär)

perf <- performance(pred, "tpr", "fpr")

plot(perf, colorize=TRUE)

Datensatz_4_3_AUC <-unlist(slot(performance(pred, "auc"),
"y.values"))

Datensatz_4_3_AUC

# AUC = 0.9475298
```

```
# Datensatz_4_4pred <- prediction(Datensatz_4_4_binominal_randomized$prediction_inner, Datensatz_4_4_binominal_randomized$Binär)

perf <- performance(pred, "tpr", "fpr")

plot(perf, colorize=TRUE)

Datensatz_4_4_AUC <- unlist(slot(performance(pred, "auc"), "y.values"))

Datensatz_4_4_AUC

# AUC = 0.9480237


# Datensatz_4_5

pred <- prediction(Datensatz_4_5_binominal_randomized$prediction_inner, Datensatz_4_5_binominal_randomized$Binär)

perf <- performance(pred, "tpr", "fpr")

plot(perf, colorize=TRUE)

Datensatz_4_5_AUC <- unlist(slot(performance(pred, "auc"), "y.values"))

Datensatz_4_5_AUC

# AUC = 0.9555402


# Datensatz_global

pred <- prediction(Hämoglobin_binominal_randomized$prediction_inner, Hämoglobin_binominal_randomized$Binär)

perf <- performance(pred, "tpr", "fpr")

plot(perf, colorize=TRUE)

Datensatz_global_AUC <- unlist(slot(performance(pred, "auc"), "y.values"))

Datensatz_global_AUC

# AUC =  0.9501817

# out-off sample performance AUC


# Gruppe_1
```

```
pred <- prediction(Gruppe1_binominal_randomized$prediction, Gruppe1_binominal_randomized$Binär)

perf <- performance(pred, "tpr", "fpr")

plot(perf, colorize=TRUE)

Gruppe1_AUC <- unlist(slot(performance(pred, "auc"), "y.values"))

Gruppe1_AUC

# AUC = 0.9329502


# Gruppe_2

pred <- prediction(Gruppe2_binominal_randomized$prediction, Gruppe2_binominal_randomized$Binär)

perf <- performance(pred, "tpr", "fpr")

plot(perf, colorize=TRUE)

Gruppe2_AUC <- unlist(slot(performance(pred, "auc"), "y.values"))

Gruppe2_AUC

# AUC =  0.9597902



# Gruppe_3

pred <- prediction(Gruppe3_binominal_randomized$prediction, Gruppe3_binominal_randomized$Binär)

perf <- performance(pred, "tpr", "fpr")

plot(perf, colorize=TRUE)

Gruppe3_AUC <- unlist(slot(performance(pred, "auc"), "y.values"))

Gruppe3_AUC

# AUC = 0.954


# Gruppe_4

pred <- prediction(Gruppe4_binominal_randomized$prediction, Gruppe4_binominal_randomized$Binär)

perf <- performance(pred, "tpr", "fpr")

plot(perf, colorize=TRUE)

Gruppe4_AUC <- unlist(slot(performance(pred, "auc"), "y.values"))
```

```
Gruppe4_AUC

# AUC = 0.9545991


# Gruppe_

pred <- prediction(Gruppe5_binominal_randomized$prediction,
Gruppe5_binominal_randomized$Binär)

perf <- performance(pred, "tpr", "fpr")

plot(perf, colorize=TRUE)

Gruppe5_AUC <- unlist(slot(performance(pred, "auc"), "y.values"))

Gruppe5_AUC

# AUC = 0.9246667

#Mittelwert der AUC und Vergleich mit globalem Modell

Diff_1_AUC = Datensatz_4_1_AUC   - Gruppe1_AUC

Diff_1_AUC

Diff_2_AUC = Datensatz_4_2_AUC  - Gruppe2_AUC

Diff_2_AUC

Diff_3_AUC = Datensatz_4_3_AUC - Gruppe3_AUC

Diff_3_AUC

Diff_4_AUC = Datensatz_4_4_AUC -  Gruppe4_AUC

Diff_4_AUC

Diff_5_AUC = Datensatz_4_5_AUC  - Gruppe5_AUC

Diff_5_AUC

Diff_data_AUC <- c(Diff_1_AUC, Diff_2_AUC, Diff_3_AUC,
Diff_4_AUC, Diff_5_AUC)

mean_AUC <- mean(Diff_data_AUC)

mean_AUC

sd_AUC <- sd(Diff_data_AUC)

sd_AUC

var_AUC <- var(Diff_data_AUC)

var_AUC

# Mittelwert_AUC =  0.005396363

# Standardabweichung_AUC = 0.01935519
```

```
# Varianz_AUC =  0.0003746233
# AUC global - Mittelwert_AUC
AUC_global_bereinigt<- Datensatz_global_AUC  - mean_AUC
AUC_global_bereinigt
# AUC_global - Mittelwert_bal_acc = 0.9447853
```
